# EMMA-STRAT: a multi-omics based machine learning framework for stratification of endometrial carcinoma molecular subtypes and MSI status

**DOI:** 10.1186/s13040-026-00587-5

**Published:** 2026-07-23

**Authors:** Naisarg Patel, Andres Salumets, Vijayachitra Modhukur

**Affiliations:** 1 Celvia CC AS, Tartu, Estonia; 2 Department of Obstetrics and Gynecology, Institute of Clinical Medicine, University of Tartu, L. Puusepa 8, Tartu, Estonia; 3 Division of Obstetrics and Gynecology, Department of Clinical Science, Intervention and Technology (CLINTEC), Karolinska Institute, and Karolinska University Hospital, Stockholm, Sweden

**Keywords:** Endometrial carcinoma, Multi-omics, Machine learning, Molecular stratification, Microsatellite instability, Supervised classification, Biomarker discovery

## Abstract

**Background:**

Uterine Corpus Endometrial Carcinoma (UCEC) is the most common gynecologic malignancy, with molecular heterogeneity influencing prognosis and treatment response. Although TCGA-defined molecular subtypes and multi-omics datasets have improved biological understanding of UCEC, externally evaluated computational frameworks for molecular stratification remain limited. To address this, we developed EMMA-STRAT, a supervised multi-omics machine learning framework integrating mRNA expression, miRNA expression, and DNA methylation data to classify UCEC genomic subtypes and microsatellite instability (MSI) status.

**Results:**

Using the TCGA cohort (*N* = 433) for model development and internal validation, we benchmarked six classifiers and evaluated final model performance on two independent Clinical Proteomic Tumor Analysis Consortium (CPTAC) cohorts (*N* = 95 and *N* = 108). Multi-omics integration consistently outperformed single-omics models, with RNA expression as the strongest standalone modality. For MSI-H versus MSS classification, a LightGBM model trained on 20 SVM-selected features per omics layer achieved an internal balanced accuracy of 98.1% and external balanced accuracies of 93.1–94.9%. For four-class genomic subtyping, a Multi-Layer Perceptron trained on 50 LASSO-selected features per omics layer achieved an internal balanced accuracy of 89.1% and external balanced accuracies of 84.7–86.2%. Both models showed favorable discrimination and probability calibration relative to reference baselines, although calibration estimates for low-prevalence classes including POLE should be interpreted cautiously. SHapley Additive exPlanations (SHAP)-based interpretability analysis identified model-selected features including *MLH1*, *CDKN2A*, *PPP4R4*, and *hsa-miR-378a*, with downstream analyses supporting their biological plausibility. All results are openly accessible via an interactive browser at https://naisarg14.github.io/EMMA-STRAT-web-viewer/index.html.

**Conclusions:**

EMMA-STRAT provides an externally evaluated, research-grade computational framework for multi-omics molecular stratification of endometrial carcinoma. Integration of mRNA, miRNA, and DNA methylation data supported prediction of MSI-H versus MSS status and TCGA-defined genomic subtypes across independent cohorts. However, since EMMA-STRAT requires multi-omics data and was not directly compared with established clinical classifiers, it should currently be interpreted as a research-oriented molecular stratification framework rather than a clinically deployable decision-making model. The developed framework provides a basis for future prospective validation, incorporation of clinicopathological variables, and direct comparison with ProMisE-based or integrated clinical risk models.

**Supplementary Information:**

The online version contains supplementary material available at 10.1186/s13040-026-00587-5.

## Introduction

Uterine Corpus Endometrial Carcinoma (UCEC) is the most common gynecologic malignancy in developed nations and remains a significant global health challenge [1, 2]. While early-stage diagnoses often correlate with favorable outcomes, a substantial subset of patients experiences aggressive disease with poor prognosis, underscoring the critical limitations of traditional histopathological classification for risk stratification [3]. The clinical heterogeneity of endometrial carcinoma is closely linked to its underlying molecular diversity, including variation in somatic mutation burden, copy-number alterations, microsatellite instability, and epigenomic regulation. Recognition of these features has led to molecular classification systems that complement histopathological assessment and improve assignment of biologically defined disease subtypes [4, 45]. Clinically oriented molecular classifiers such as Proactive Molecular Risk Classifier for Endometrial Cancer (ProMisE) have translated these molecular insights into a practical framework using surrogate markers, including mismatch repair immunohistochemistry, POLE mutation testing, and p53 assessment, to support risk stratification in endometrial carcinoma.

The Cancer Genome Atlas (TCGA) Research Network made a major contribution by conducting a comprehensive multi-omics analysis that classified EC into four molecularly distinct subtypes: (1) POLE ultramutated, which is linked to an exceptionally favorable prognosis; (2) microsatellite instability-high (MSI-H) hypermutated, associated with an intermediate prognosis; (3) copy-number low (CNV-Low), also linked to an intermediate prognosis; and (4) copy-number high (CNV-High), which is associated with the poorest clinical outcomes [4, 5]. These subtypes are defined by their distinct patterns of somatic mutation burden, copy-number alterations, and microsatellite instability. This molecular subtyping framework has refined prognostic stratification beyond histopathology by linking biologically distinct tumor groups with clinical outcome and treatment-relevant biomarkers, including mismatch repair deficiency and MSI-H status, thus fundamentally transforming the diagnostic and therapeutic landscape of EC [6, 7].

Furthermore, testing for mismatch repair (MMR) status and microsatellite instability (MSI) in endometrial carcinoma carries significant diagnostic, prognostic, and therapeutic implications. MMR deficiency (MMRd/MSI) serves as an important diagnostic marker for endometrioid-type endometrial carcinoma, aids in identifying patients at increased risk for Lynch syndrome, provides prognostic information and predicts potential responsiveness to immune checkpoint inhibitor therapies [8, 9]. Consequently, the International Society of Gynecological Pathologists (ISGyP) recommends universal MMR/MSI testing for all endometrial carcinoma cases regardless of patient age [10]. Currently, MSI detection in clinical practice primarily relies on polymerase chain reaction (PCR)-based assays and immunohistochemistry (IHC) for mismatch repair proteins. These established methods require dedicated laboratory workflows and specialized expertise, and their availability may vary across institutions [11]. Molecular stratification further reveals distinct prognostic groups: *POLE*-mutated tumors associated with excellent outcomes and p53-abnormal tumors linked to poor prognosis, while MMR-deficient and non-specific molecular profile (NSMP) tumors show intermediate clinical behavior. However, molecular surrogates such as p53 immunohistochemistry do not perfectly capture underlying TP53 mutation status, and a subset of high copy-number tumors lack detectable TP53 mutations. These limitations highlight the importance of integrating molecular and pathological information for more accurate classification and risk stratification [12].

Recent advancements in computational algorithms have resulted in the development of tools like MSIsensor and MANTIS, which analyze genome-wide sequencing data to produce instability scores [13, 14]. However, these approaches can be resource-intensive, and their accuracy may vary depending on sequencing quality, coverage, and tumor purity. Moreover, they primarily detect somatic alterations at microsatellite loci which are the downstream consequences of MSI, rather than directly assessing mismatch repair (MMR) deficiency, the underlying biological cause of MSI. Alternative strategies have leveraged deep learning models to predict MSI status from whole-slide histopathology images, but such approaches typically require high-quality immunostained slides and standardized imaging protocols, which may limit scalability [15, 16]. Other studies have trained models using transcriptomic data, including bulk RNA sequencing, microarrays, and single-cell expression profiles; however, these models often lack systematic optimization for cross-cohort generalizability and external validation [17, 18]. Broader attempts at molecular subtype discovery have relied on unsupervised clustering, which tends to produce groupings that are difficult to translate into clinically actionable classifiers and cannot be directly applied to newly diagnosed patients [19, 20].

Determination of the broader genomic subtypes is even more complex, as classification requires integration of MSI status, *POLE* mutation status, and copy-number variation (CNV) profiles to assign a final molecular category [21, 22]. Traditionally, this multi-layered characterization relies on whole-genome or comprehensive sequencing approaches. More recently, computational models based on whole-slide histopathology images have also been developed to infer these subtypes [23, 24]. Other studies have attempted patient stratification into prognostic risk groups using Cox regression–based scores or unsupervised clustering [25, 26]. However, the TCGA-defined molecular subtypes remain among the most established predictors of clinical outcomes and prognosis.

Existing multi-omics frameworks differ substantially in design and intended use. DIABLO and MOFA + are R/Bioconductor packages oriented toward biomarker discovery and unsupervised dimensionality reduction, requiring substantial deployment as supervised classifiers for predefined clinical labels [27, 28]. *Flexynesis* offers supervised multi-omics learning but has not been evaluated for endometrial cancer subtyping with independent external validation [29]. EMMA-STRAT addresses these gaps through three design principles: task-specific feature selection benchmarking across four methods and six feature counts per omics layer; systematic comparison of six architecturally diverse classifiers including a graph neural network; and mandatory external validation on two independent cohorts as a primary design criterion rather than a post-hoc step. 

**Fig. 1:**
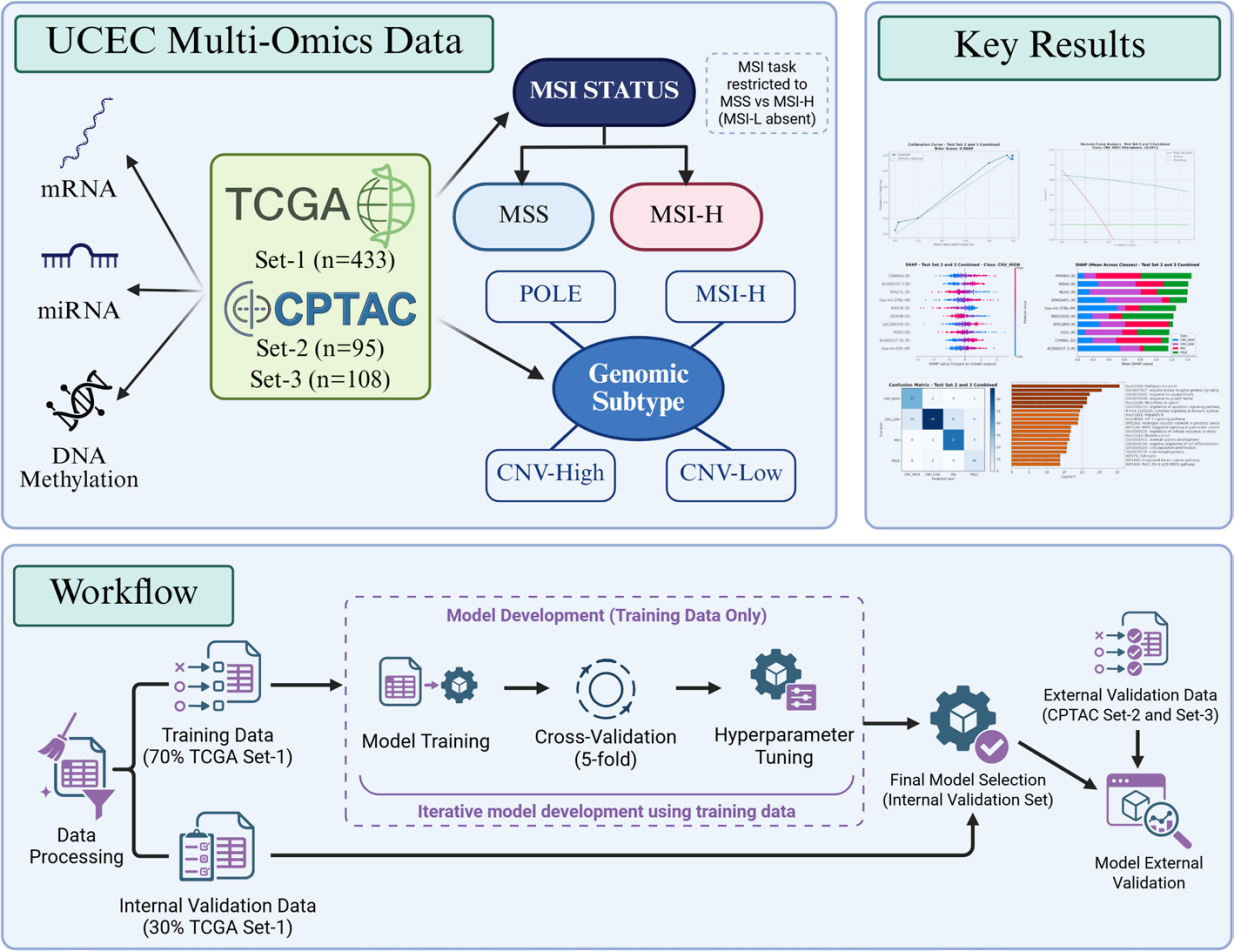
Overview of the EMMA-STRAT framework. EMMA-STRAT integrates mRNA expression, miRNA expression, and DNA methylation data from TCGA and two independent CPTAC cohorts within a supervised machine learning workflow for MSI-H versus MSS classification and molecular subtype classification in uterine corpus endometrial carcinoma. The framework includes data preprocessing, cohort harmonization, task-specific feature selection, model training, internal validation, independent external evaluation, and interpretation of model-selected molecular features. MSI-L cases were absent from the available cohort annotations; therefore, the MSI classification task was restricted to the binary distinction between MSI-H and MSS tumors, and MSI-L tumors were not represented during training, internal validation, or external validation

Although comprehensive multi-omics profiling is not yet routinely available in many clinical laboratories, EMMA-STRAT is intended for research settings in which transcriptomic, miRNA, and DNA methylation data are available, rather than as a replacement for established PCR- or immunohistochemistry-based MSI/MMR testing. In summary, we developed and evaluated a supervised multi-omics machine learning framework to stratify UCEC genomic subtypes and MSI-H versus MSS status (Fig. 1). The framework was designed to address two related but distinct prediction tasks: binary MSI-H versus MSS classification, reflecting the diagnostic and therapeutic relevance of MSI/MMR status, including Lynch syndrome screening and identification of patients who may benefit from immune checkpoint inhibitor therapy [59], and four-class genomic subtype classification, reflecting broader TCGA-defined molecular stratification across POLE, CNV-High, CNV-Low, and MSI subtypes [4, 12]. Our approach integrates miRNA expression, mRNA expression, and DNA methylation data, benchmarks single-omics versus multi-omics models and evaluates model performance on independent CPTAC cohorts using harmonized preprocessing. Accordingly, EMMA-STRAT should be interpreted as a research-oriented molecular stratification framework, and its incremental value beyond established clinical classification approaches, including ProMisE, MMR-IHC/MSI testing, POLE sequencing, p53-IHC, and integrated clinicopathological models, remains to be established.

## Methodology

### Data extraction and preprocessing

Data for UCEC tissue samples were obtained from The Cancer Genome Atlas (TCGA) and the Clinical Proteomic Tumor Analysis Consortium (CPTAC) [22, 30]. Metadata including sample ID, sample type, and subtype for TCGA samples were obtained from cBioPortal, while corresponding data for CPTAC and independent CPTAC cohorts were retrieved from the LinkedOmics database for the Uterine Corpus Endometrial Carcinoma (UCEC) cancer type [31, 32]. Raw count files for miRNA and RNA sequencing data, as well as beta values for CpG methylation, were downloaded from the Genomic Data Commons (GDC) portal (Data Release 44.0) using custom Python scripts via the GDC API [33].

All datasets were preprocessed according to data type and labeled based on sample IDs to generate the final CSV files used for downstream analyses. For miRNA data, miRNA identifiers and read counts were extracted, counts per million (CPM) were calculated, and miRNAs with CPM < 1 in more than 95% of samples were removed. The remaining values were log-transformed using log(CPM + 1) normalization. For RNA sequencing data, gene identifiers and transcripts per million (TPM) values were extracted, and genes with TPM < 1 in more than 95% of samples were excluded, followed by log(TPM + 1) normalization. For DNA methylation data, CpG probe identifiers and beta values were extracted, and Illumina annotations were used to map probes to corresponding gene IDs. Genes with variance < 0.01 were filtered out, and beta values were converted to M-values for model training [34].

### Data processing and cohort definition

Following preprocessing and cohort harmonization, samples were matched across omics layers by sample ID, and only samples with complete mRNA, miRNA, and DNA methylation profiles were retained for downstream modelling. Throughout this study, the TCGA, CPTAC, and independent CPTAC datasets are denoted as Set-1, Set-2, and Set-3, respectively. The three datasets were compared and only features common to all datasets were retained. Sample identifiers were cross-checked to ensure that there was no overlap among the three sets, and only samples with complete multi-omics data were retained for subsequent analyses. Set-1 (TCGA) was further randomly divided into 70% training and 30% internal validation subsets using the *train_test_split* function from *scikit-learn* [36], referred to as the Training Set and Internal Validation Set, respectively. The Training Set was used for model development and hyperparameter tuning, whereas the Internal Validation Set was used for final model selection. Set-2 and Set-3 served as independent external validation cohorts.

Principal Component Analysis (PCA) and t-distributed Stochastic Neighbor Embedding (t-SNE) analyses revealed substantial batch effects across datasets. Therefore, batch correction was performed using the *pyComBat* implementation available in the *InMoose* Python library [35]. To prevent information leakage, the Training Set was designated as the reference batch (*ref_batch*), and the Internal Validation Set, Set-2, and Set-3 were independently adjusted relative to this reference. Consequently, the batch correction parameters were estimated exclusively from the Training Set, ensuring that no information from the validation or external cohorts influenced model development. This strategy preserved the independence of Set-2 and Set-3.

Within this cohort structure, two related but distinct classification tasks were evaluated. The first was binary MSI-H versus MSS prediction, using samples with available MSI status labels. The second was four-class genomic subtype prediction, using samples annotated as POLE, CNV-High, CNV-Low, or MSI. For genomic subtype and microsatellite instability (MSI) status analyses, samples with missing (NA) labels were excluded from the corresponding task only. Additionally, for MSI status prediction, the clinical annotations linked to the multi-omics data did not contain any MSI-L samples. The model was therefore trained and validated exclusively as a binary MSI-H versus MSS classifier and cannot assign an MSI-L label. MSI-L tumors, if present in future cohorts, were not represented during training or validation, and model performance for such cases therefore remains unknown.

### Feature selection

Four feature selection methods. ANOVA, Random Forest (RF), Support Vector Machine (SVM), and LASSO, were applied on the training set to identify the top 10, 20, 50, 100, 150, and 200 features from each omics layer. ANOVA was selected for its simplicity and effectiveness as a variance-based filtering method. Random Forest was chosen due to its tree-based ensemble structure and ability to capture nonlinear feature interactions. SVM-based feature selection was employed for its strong discriminative power in high-dimensional spaces. LASSO was included because of its embedded regularization mechanism, which performs feature selection by shrinking irrelevant feature coefficients to zero. All methods except ANOVA applied a Recursive Feature Elimination (RFE) technique to iteratively rank and eliminate less informative features. RFE operates by repeatedly training the model, evaluating feature importance, and removing the least contributing features at each step until the desired number of features is retained. This wrapper-based approach enables RF, SVM, and LASSO to identify compact and highly discriminative feature subsets while accounting for feature interactions and model performance. The *RFE*, *LinearSVC*, *RandomForestClassifier*, *LogisticRegression* and *SelectKBest* functions from *scikit-learn* were used for the feature selection. To prevent data leakage, all feature selection procedures were applied exclusively to the training set (70% of Set-1). The validation set (30% of Set-1) and external sets (Set-2, Set-3) were not accessed at any stage of feature selection. Feature counts (10, 20, 50, 100, 150, 200) were evaluated using 5-fold cross-validation performance on the training set.

For single-omics models, the selected feature counts refer to the number of features retained from each corresponding omics layer. For multi-omics models, feature selection was performed independently within each omics layer, after which the selected features were subsequently concatenated into a single input matrix. Thus, using the top 10, 20, 50, 100, 150, or 200 features per omics layer resulted in total multi-omics input dimensions of 30, 60, 150, 300, 450, and 600 features, respectively. Throughout the study, the multi-omics model nomenclature refers to the number of features selected per omics layer rather than the total of concatenated feature count, to maintain consistency with the single-omics analyses.

### Single-omics models

The selected features were used to train K-Nearest Neighbors (KNN), Random Forest (RF), Support Vector Machine (SVM), Light Gradient Boosting Machine (LGBM), Multi-Layer Perceptron (MLP), and Graph Neural Network (GNN) models on the training split from Set-1. The hyperparameters for all models were optimized using the *Optuna* library [37]. Model-specific hyperparameters and their corresponding search ranges were defined according to the characteristics of each algorithm and prior methodological considerations. 5-fold CV was used while training the models to remove any bias. Detailed model construction and training details can be found in Supplementary Methods 1.1–1.6. The models were evaluated on the 30% validation set from Set-1. The *scikit-learn*,* lightgbm and tensorflow* libraries in Python were used to train the models [38]. The models were evaluated on the validation set using balanced accuracy, F1-score, and area under the receiver operating characteristic (ROC) curve (AUC) [39]. Additionally, confusion matrices were examined to analyze misclassification patterns.

### Multi-omics models

Additional models were developed using an integrated multi-omics approach. Features from all three omics datasets were combined into a single input matrix and used for model training. The selected feature subsets from each omics layer (mRNA, miRNA, and DNA methylation) were horizontally concatenated along the feature axis into a single sample × feature matrix, with no inter-layer weighting or transformation applied. The concatenated matrix was standardised using a single *StandardScaler* fit on the training partition and applied unchanged to the validation and external sets. This early integration strategy was chosen for its simplicity and interpretability, and to ensure that any performance gain over single-omics models reflects complementary information across layers rather than architectural complexity. The same *Optuna*-based hyperparameter optimization and five-fold cross-validation strategy were applied to ensure consistency and fair performance evaluation.

### External validation

Sets 2 and 3 were used as external validation datasets. Following Ho et al. [20], convergent validation was applied by evaluating multiple model configurations across feature selection methods on each external cohort, enabling identification of consistently high-performing feature sets. Divergent validation was applied by testing the top-performing models on both Set-2 and Set-3 independently, assessing the generalisability of the selected feature sets across two cohorts with distinct sample compositions and MSI determination methodologies. These datasets were scaled and label-encoded using the same scaler and labeling function as the training set, which is stored to ensure reproducibility. The selected feature subset was applied, and the trained models were used to generate predictions. The model performance was evaluated using balanced accuracy, F1-score, and area under the ROC curve (AUC). To quantify uncertainty in all reported performance estimates, bootstrapped 95% confidence intervals were computed for balanced accuracy, macro F1-score, and AUC across all evaluation sets [40]. For each model and dataset, 10,000 bootstrap samples were drawn with replacement from the predictions and true labels, and metrics were recomputed on each resample. The 2.5th and 97.5th percentiles of the resulting distribution were used as the lower and upper confidence interval bounds respectively.

### Calibration curves

Calibration curves, also known as reliability diagrams, are used to assess how well the probabilistic predictions of a binary classifier are calibrated. They compare predicted probabilities with observed outcome frequencies across different probability bins. Calibration curves were constructed using the *calibration_curve* function implemented in *scikit-learn*, with predictions grouped into 10 equally spaced bins (n_bins = 10). Model calibration was further evaluated using the Brier score (Eq. 1), which measures the mean squared difference between predicted probabilities and actual outcomes. The *brier_score_loss* function from *scikit-learn* was used to compute this metric, with lower values indicating better calibration and overall predictive accuracy [41]. For multi-class, the calibration curve is generated using a one-vs rest classification using threshold probability *scikit-learn*.

1 $$\:BS(Y,\widehat{P})\:=\:\frac{1}{N}{\sum\:}_{i=0}^{N-1}{\sum\:}_{k=0}^{K-1}({y}_{i,k}-{\widehat{p}}_{i,k}{)}^{2\:}$$

Where *N* is the total number of samples, *K* is the number of classes, $\:{y}_{i,k}$ is a binary indicator for the true class, and $\:{\widehat{p}}_{i,k}$ is the predicted class probability for sample i.

To provide a reference for model calibration, a no-skill classifier was defined as one that assigns the marginal class prevalence as the predicted probability for all samples. For binary outcomes, the corresponding no-skill Brier score is given by * p(1-p)*, where *p* denotes the class prevalence. For multiclass outcomes, the no-skill mean Brier score was calculated as the average of the per-class no-skill scores derived from the subtype prevalences in the combined external test set. Calibration performance was interpreted relative to both this no-skill baseline and the perfectly calibrated reference line.

Expected Calibration Error (ECE) and Maximum Calibration Error (MCE) were computed for both models across all evaluation sets using top-label confidence with 10 equal-width bins. ECE was defined as the weighted average absolute difference between mean predicted confidence and observed accuracy across non-empty bins, whereas MCE represented the maximum such difference. Models with a validation-set ECE greater than the pre-specified threshold of 0.05 were considered poorly calibrated. For these models, post-hoc temperature scaling was performed by optimizing a single scalar temperature parameter (T) to minimize the negative log-likelihood on the internal validation set logits using *SciPy’s* bounded scalar minimization. The fitted temperature was subsequently applied to the logits of all evaluation sets before softmax transformation, without any further fitting on external data.

### Decision curve

Decision Curve Analysis was used to evaluate model net benefit across threshold probabilities ranging from 0 to 1, using treat-all and treat-none strategies as reference comparators. At each threshold, predicted probabilities were converted into binary classifications, and net benefit was calculated by balancing true positives against false positives [42]. Net benefit was computed as shown in Eq. 2.

2 $$\:Net\:Benefit\:=\:\frac{TruePositiveCount}{n}\:-\:\frac{FalsePositiveCount}{n}\left(\frac{{p}_{t}}{1-{p}_{t}}\right)$$

Where *n* is the sample size, and $\:{p}_{t}$ is the threshold probability.

The model’s performance was compared with treat-all and treat-none strategies. Decision curve analysis was conducted using subtype-specific threshold probability ranges reflecting molecular risk stratification: POLE (0.05–0.30), MSI-H (0.10–0.50), CNV-Low (0.15–0.60), and CNV-High (0.20–0.50), consistent with published TCGA and clinical validation studies [22, 43–45]. Because net benefit in decision curve analysis is a prevalence-weighted quantity, with the false-positive penalty scaled by $\:\left(\frac{{p}_{t}}{1-{p}_{t}}\right)$, the threshold ranges and interpretation of net benefit were informed by the observed subtype prevalences in the study cohort (POLE: 6.0%, CNV-High: 18.1%, MSI-H: 28.6%, and CNV-Low: 47.2%).

### SHAP

Feature importance was assessed using SHAP (SHapley Additive exPlanations), using the *SHAP Explainer* to calculate feature importance and *summary_plot* function generating beeswarm and bar plots to highlight the top features contributing to the predictions for each cancer type [46]. SHAP values were computed for each feature across all samples on a per-class basis using Eq. 3. Class-specific SHAP values were then aggregated across classes to derive an overall importance score for each feature, as defined in Eq. 4.

3 $$\:{I}_{f,c}=\:\frac{1}{N}{\sum\:}_{i=1}^{N}\left|{SHAP}_{i,f,c}\right|$$

Where $\:{I}_{f,c}$ denotes the mean absolute SHAP value of feature * f* for class c, computed across all * N*samples; $\:{SHAP}_{i,f,c}$ represents the contribution of feature * f* to the prediction of class * c* for sample * i*.

4 $$\:{Importance}_{f}=\:\frac{1}{C}{\sum\:}_{c=1}^{C}\frac{1}{N}{\sum\:}_{i=1}^{N}\left|{SHAP}_{i,f,c}\right|$$

where $\:{Importance}_{f}$ represents the global importance of feature * f*, computed as the mean absolute SHAP value across all samples (* N*) and all classes (* c*). The term $\:{SHAP}_{i,f,c}$ denotes the contribution of feature * f* to the prediction of class c for sample * i*.

### Survival analysis

Survival analyses were performed for the top genes using *kaplan_meier_estimator* from *scikit-survival* [47]. The samples were stratified into high- and low-expression groups for each gene using the median expression value as a cutoff. Kaplan–Meier survival curves were estimated based on event status and follow-up time. Adjusted hazard ratios (HRs) were obtained from a multivariable Cox proportional hazards model fitted with the *CoxPHFitter* function from the *lifelines* library [48], and the corresponding HR is annotated on each curve. Survival probability was plotted against follow-up time. A detailed description of the Cox regression, including the covariates included and the procedures used to evaluate model assumptions, is provided in Supplementary Methods S2.

### Downstream analysis

The miRNA and RNA features selected from the top-performing models were used for downstream functional analysis. Overlap analysis was performed between the SVM-based 20-feature set (MSI model) and the LASSO-based 50-feature set (genomic subtype model) to identify shared model-selected features.

miRNA precursors were converted to their corresponding mature miRNA forms prior to target analysis. Validated miRNA–target interactions were retrieved using *mirTargetLink 2.0* [49]. The validated target genes were then used as input for functional enrichment analysis in *Metascape*, which generated Gene Ontology (GO) enrichment bar plots [50]. Additionally, gene identifiers were converted to protein IDs using *g: Profiler*, and the resulting protein list was submitted to STRING to construct the protein–protein interaction (PPI) network [51, 52]. The reference network was based on the Homo sapiens STRING database using the full STRING interaction network. Interactions with a confidence score of at least 0.400 (medium confidence) were retained, and network enrichment analyses were performed using a false discovery rate (FDR) stringency of 5%.

To assess the biological relevance of the model-selected DNA methylation features, cross-omics methylation–RNA correlation analysis was performed by testing associations between gene-level methylation M-values and matched RNA expression values for genes with detectable expression. Correlations were adjusted for multiple testing using the Benjamini–Hochberg false discovery rate procedure, and inverse associations were interpreted as consistent with methylation-associated transcriptional repression.

### Benchmarking against existing multi-omics frameworks

To contextualise the performance of EMMA-STRAT relative to existing multi-omics supervised learning frameworks, *Flexynesis* [29] was applied to the same UCEC dataset using three of its available integration strategies: *DirectPred*, supervised variational autoencoder (*supervised_vae*), and cross-modal prediction (*CrossModalPred*). The benchmark was designed such that the two frameworks differed only in their modeling strategies. Specifically, *Flexynesis* was provided with the same post-quality-control and normalized mRNA, miRNA, and DNA methylation matrices, as well as the same Set-1, Set-2, and Set-3 sample partitions used by EMMA-STRAT. External cohorts were reserved exclusively for evaluation and were not accessed during model fitting or optimization.

Both frameworks were operated using their native optimization procedures. EMMA-STRAT employed task-specific feature selection benchmarking and *Optuna*-based hyperparameter optimization, whereas *Flexynesis* utilized its built-in variance filtering and Laplacian-score-based feature reduction together with automated hyperparameter optimization, with all native procedures enabled. Performance was evaluated on the same external validation cohorts (Set-2 and Set-3) using balanced accuracy and macro F1-score as the primary metrics. Identical evaluation scripts and label-handling procedures were applied to both frameworks to ensure a consistent comparison. Detailed configuration settings, modality-specific feature retention counts, and cohort-wise performance results are provided in Supplementary Methods S3.

### Interactive results browser

To facilitate exploration of EMMA-STRAT model performance, an interactive web-based results browser was developed and deployed via GitHub Pages. The webpage provides a tabular view of all evaluated model configurations, filterable by omics type, classification task, model architecture, and feature selection method, alongside visualisations of performance metrics including balanced accuracy, macro F1-score, and AUC across internal and external evaluation sets.

## Results

### Dataset summary

The data were downloaded and processed, and the dimensional changes during data extraction and preprocessing are summarized in Supplementary Table S1. The final integrated dataset comprised 2,576 gene-level DNA methylation features, 503 miRNA features, and 27,366 mRNA features. A summary of datasets from Set-1, Set-2, and Set-3 is presented in Table 1. Batch effects were observed in the mRNA and miRNA datasets and were reduced using the *pyComBat* library (Supplementary Figure S2).

**Table 1:** Distribution of MSI status and genomic subtypes across Set-1, Set-2, and Set-3 cohorts

Characteristic	Set-1 (TCGA) Training + internal validation *N* = 433	Set-2 (CPTAC) External validation *N* = 95	Set-3 (CPTAC) External validation *N* = 108
**Clinical and demographic**			
Age at diagnosis, years Mean ± SD	63.8 ± 11.3	63.4 ± 10.3	64.4 ± 10.0
**Histological subtype**			
Endometrioid	315 (72.7%)	80 (84.2%)	92 (85.2%)
Serous	97 (22.4%)	12 (12.6%)	13 (12.0%)
Other / Mixed	21 (4.8%)	2 (2.1%)	3 (2.8%)
Unknown / missing	0 (0.0%)	1 (1.1%)	0 (0.0%)
**International Federation of Gynecology and Obstetrics (FIGO) stage**			
Stage I	237 (54.7%)	70 (73.7%)	67 (62.0%)
Stage II	36 (8.3%)	7 (7.4%)	10 (9.3%)
Stage III	89 (20.6%)	14 (14.7%)	15 (13.9%)
Stage IV	19 (4.4%)	3 (3.2%)	6 (5.6%)
Unknown / missing	52 (12.0%)	1 (1.1%)	10 (9.3%)
**Tumor grade**			
Grade 1	60 (13.9%)	35 (36.8%)	29 (26.9%)
Grade 2	89 (20.6%)	37 (38.9%)	53 (49.1%)
Grade 3	284 (65.6%)	8 (8.4%)	24 (22.2%)
Unknown / missing	0 (0.0%)	15 (15.8%)	2 (1.9%)
**MSI status classification labels**			
MSS	277 (64.0%)	73 (76.8%)	72 (66.7%)
MSI-H	126 (29.1%)	21 (22.1%)	36 (33.3%)
Missing / excluded	30 (6.9%)	1 (1.1%)	0 (0.0%)
Total N for MSI task (labeled)	403	94	108
**Genomic subtype classification labels**			
CNV-HIGH	144 (33.3%)	22 (23.2%)	14 (13.0%)
CNV-LOW	104 (24.0%)	44 (46.3%)	50 (46.3%)
POLE	39 (9.0%)	7 (7.4%)	5 (4.6%)
MSI	109 (25.2%)	21 (22.1%)	36 (33.3%)
Missing / excluded	37 (8.5%)	1 (1.1%)	3 (2.8%)
Total N for genomic-subtype task (labeled)	396	94	105
**Omics data availability**			
mRNA features; post-QC (per cohort)	27,892	43,787	45,961
miRNA features; post-QC (per cohort)	619	666	617
DNA methylation features; post-QC (per cohort)	4158	4123	8030
mRNA features; harmonized (used in models)	27,366	27,366	27,366
miRNA features; harmonized (used in models)	503	503	503
DNA methylation features; harmonized (used in models)	2576	2576	2576
MSI determination method	7-marker PCR panel (TCGA)	MSIsensor (genomic, CPTAC)	MSIsensor (genomic, CPTAC)

Values are reported as n (%). Percentages are calculated using the total cohort size for each dataset

### Feature selection

The features used for model development are provided in Supplementary Table S2 for the MSI model (SVM-based feature selection, 20 features for each omics) and Supplementary Table S3 for the genomic subtype model (LASSO-based feature selection, 50 features for each omics). Overlap analysis demonstrated partial concordance between the genomic subtype and MSI models (Supplementary Fig. 1). Across all features, 30 markers were shared. RNA features showed the highest overlap (17 shared), whereas miRNA (8 shared) and methylation features (5 shared) exhibited more subtype-specific patterns. These findings show that the two classification tasks share a subset of selected features, while also retaining task-specific feature patterns that likely reflect differences in the statistical signals required for MSI status prediction and four-class genomic subtype classification. The supplementary tables include corresponding gene symbols for each RNA feature, as the original data were annotated using Ensembl identifiers. In addition, selected feature files for all feature selection methods, numbers of features, and molecular subtypes are available in the *feature_selection* folder of the project’s GitHub repository.

### Single-omics models

Single-omics models were evaluated across RNA expression, DNA methylation, and miRNA expression for both tasks. RNA expression consistently yielded the strongest performance. Model ranking and final configuration selection were based exclusively on performance on the internal validation set (30% of Set-1). The external cohorts (Set-2 and Set-3) were reserved solely for independent evaluation and were not used during model selection, hyperparameter optimization, or any stage of model development. The external datasets were not used for model selection and served only as independent evaluation sets. For MSI classification, the top RNA configuration (MLP, SVM, 100 features) achieved an internal macro F1-score of 0.98 and external macro F1-score of 0.86, with average external balanced accuracy of 89.0%; remaining top configurations achieved external macro F1-scores of 0.83–0.87 and average external balanced accuracies of 86.4–91.0% (Supplementary Table S4). For genomic subtype classification, MLP with LASSO and 100 features achieved the highest internal macro F1-score of 0.87, with an external macro F1-score of 0.73 and average external balanced accuracy of 75.7% (Supplementary Table S5).

DNA methylation achieved moderate performance across both tasks. For MSI classification, the top configuration (MLP, SVM, 150 features) achieved an internal macro F1-score of 0.95 and external macro F1-score of 0.82, with average external balanced accuracy of 83.3%; remaining top configurations achieved external macro F1-scores of 0.76–0.86 and average external balanced accuracies of 78.6–87.2%. For genomic subtype classification, MLP with LASSO and 150 features achieved the highest internal macro F1-score of 0.81, with an external macro F1-score of 0.66 and average external balanced accuracy of 68.4%, with SVM (LASSO, 200 features) achieving the strongest external generalisation (external macro F1-score: 0.76; average external balanced accuracy: 78.7%), reflecting the more dispersed distribution of epigenetic changes requiring larger feature sets (Supplementary Table S5).

miRNA expression yielded the lowest standalone performance across both tasks. For MSI classification, the top configuration (GNN, SVM, 50 features) achieved an internal macro F1-score of 0.89 and external macro F1-score of 0.72, with average external balanced accuracy of 71.9%; remaining top configurations achieved external macro F1-scores of 0.70–0.76 and average external balanced accuracies of 71.8–78.2%. For genomic subtype classification, GNN with LASSO and 150 features achieved the highest internal macro F1-score of 0.75, with an external macro F1-score of 0.62 and average external balanced accuracy of 66.6%, with remaining top configurations achieving external macro F1-scores of 0.56–0.63 (Supplementary Table S5). The consistent performance hierarchy of RNA > DNA methylation > miRNA, and the substantially lower accuracies for genomic subtyping relative to MSI classification, motivated development of an integrated multi-omics approach.

**Fig. 2:**
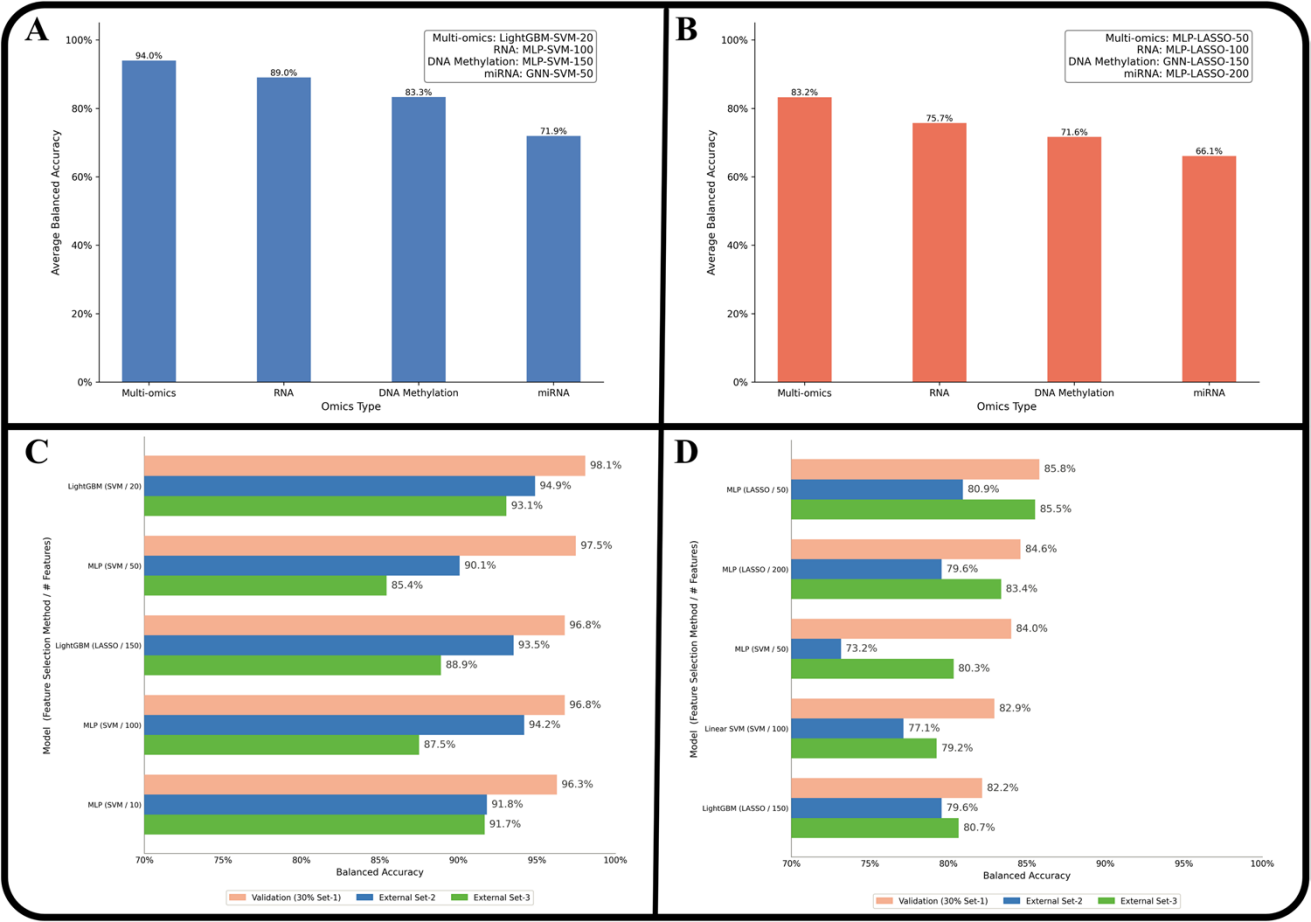
Multi-omics cancer classification performance across data types and model configurations. Average balanced accuracy across four omics types (Multi-omics, RNA, DNA Methylation, miRNA) for the top-performing model combination on the (**A**) MSI Status and (**B**) Genomic Subtype. Balanced accuracy of the top 5 model–feature selection–feature count combinations for (**C**) MSI Status and (**D**) Genomic Subtype, evaluated on the validation set (30% Set-1), External Set-2, and External Set-3. Models are ranked by validation performance

### Multi-omics models

Multi-omics integration consistently outperformed all single-omics models across both classification tasks. For MSI status classification, the integrated approach achieved an average external balanced accuracy of 94.0%, representing a gain of approximately 5% points over the best single-omics layer (RNA, 89.0%). For genomic subtype classification, the multi-omics model reached 85.4% average external balanced accuracy, surpassing the best single-omics result (RNA, 75.7%) by 7.5% points (Fig. 2A and B). Similar to the single-omics models, model selection was based solely on validation-set performance. The external datasets were not involved in the selection process and were used only for evaluation. Although concatenation of features from multiple omics layers increased the overall dimensionality of the integrated models, the multi-omics approach consistently outperformed single-omics models with comparable or larger numbers of selected features, suggesting that the performance gains were attributable to complementary biological information rather than feature quantity alone. While increased dimensionality may raise concerns regarding overfitting, the selected models showed consistent performance across two independent external validation cohorts, supporting the stability of the observed performance under the evaluated cohort settings.

For MSI versus MSS classification, the LightGBM model trained on 20 SVM selected features achieved the best performance, with an internal macro F1-score of 0.98, average external macro F1-score of 0.93, and average external balanced accuracy of 94.0%. The next strongest configurations, MLP (SVM, 100 features) and LightGBM (LASSO, 150 features) also achieved average external balanced accuracies of 86.0% and 91.2% respectively, with external macro F1-scores of 0.83 and 0.89 (Supplementary Table S6).

For genomic subtype classification, the MLP model trained on 50 LASSO-RFE-selected features achieved the best performance, with an internal macro F1-score of 0.87, average external macro F1-score of 0.79, and average external balanced accuracy of 85.4%. Other notable top configurations, MLP (LASSO, 100 features) and MLP (LASSO, 150 features), achieved average external balanced accuracies of 82.1% and 78.9% with external macro F1-scores of 0.80 and 0.74 respectively (Supplementary Table S7).

### External validation

Performance estimates with bootstrapped 95% confidence intervals (*n* = 10,000 resamples) are reported for both top-performing models across all evaluation sets and are summarised in Table 2.

**Table 2:** Performance of top-performing models across evaluation sets with bootstrapped 95% confidence intervals (*n* = 10,000 resamples)

MSI Status (LGBM Model - SVM FS [20])						
Evaluation Set	Balanced Accuracy		AUC		Macro F1	
	Mean	95% CI	Mean	95% CI	Mean	95% CI
Internal (30% Set-1)	98.1%	94.7%–100.0%	1.00	0.99–1.00	98.1%	95.0%–100.0%
External Set-2	94.9%	88.7%–99.3%	0.96	0.87–1.00	92.7%	85.4%–98.4%
External Set-3	93.1%	87.3%–97.8%	0.96	0.93–0.99	92.8%	86.9%–97.1%

Genomic Subtype (MLP Model - LASSO FS [50])						
Evaluation Set	Balanced Accuracy		AUC		Macro F1	
	Mean	95% CI	Mean	95% CI	Mean	95% CI
Internal (30% Set-1)	89.1%	82.3%–94.8%	0.96	0.92–0.99	87.2%	79.6%–93.5%
External Set-2	86.2%	76.3%–93.7%	0.96	0.93–0.99	83.4%	73.0%–91.2%
External Set-3	84.7%	72.6%–92.7%	0.95	0.88–0.99	75.5%	64.4%–84.9%

**Fig. 3:**
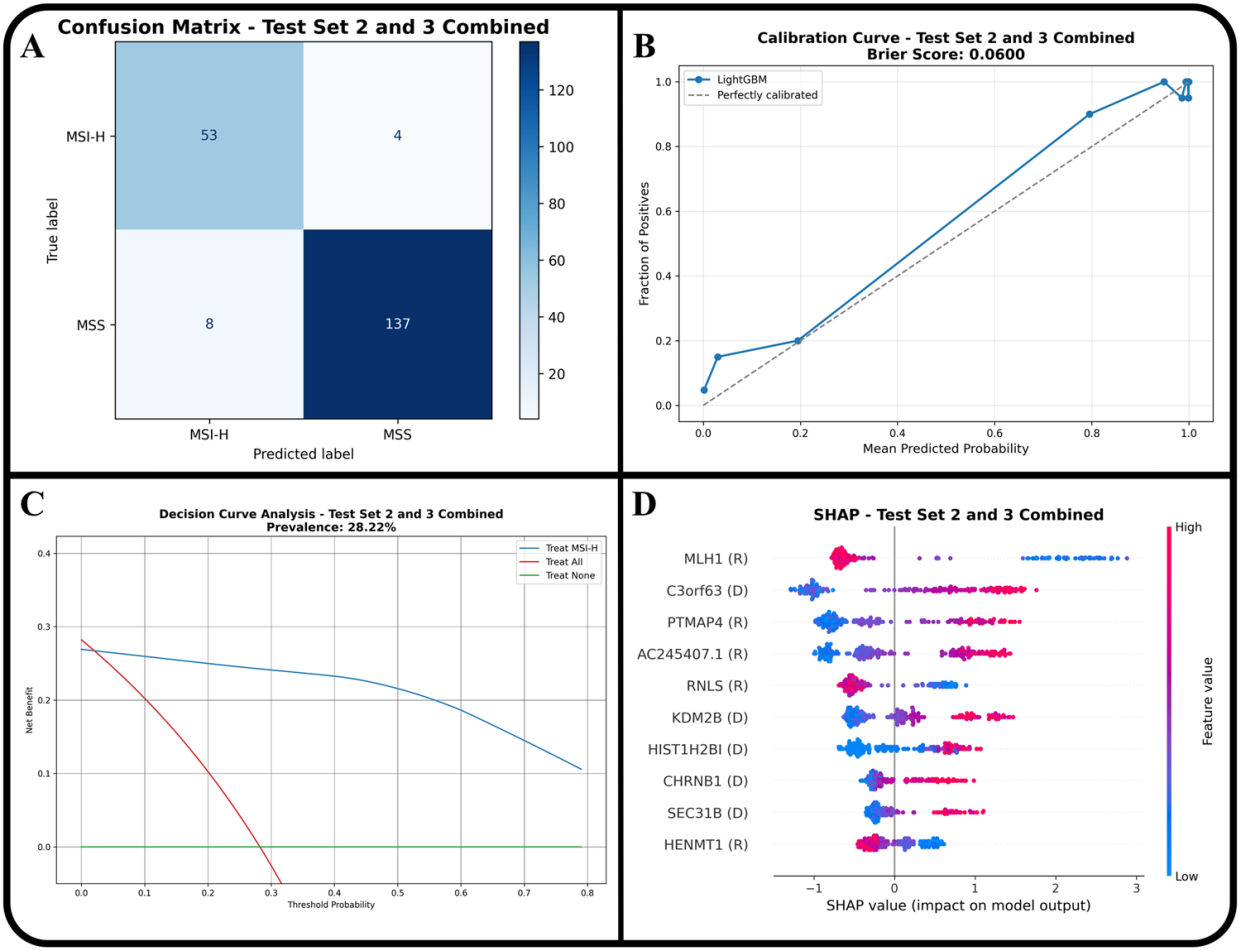
Performance and interpretability of the LightGBM model on combined test sets. (**A**) Confusion matrix showing classification performance of the model for MSI-H and MSS samples. (**B**) Calibration curve illustrating agreement between predicted probabilities and observed outcomes. (**C**) Decision curve analysis demonstrating the net benefit of the model compared with treat-all and treat-none strategies. (**D**) SHAP summary plot highlighting the most important features and their impact on model predictions

The LightGBM model for MSI-H versus MSS classification showed higher performance across all three evaluation sets (Fig. 3A). On the internal validation set, the model achieved a balanced accuracy of 98.1%, AUC of 1.00, and macro F1-score of 98.1%. On Set-2, the model attained a balanced accuracy of 94.9%, AUC of 0.96, and macro F1-score of 92.7%. On Set-3, it achieved a balanced accuracy of 93.1%, AUC of 0.96, and macro F1-score of 92.8%. The upper CI bound of 1.00 for Set-2 AUC reflects the limited MSI-H sample size in that cohort (*n* = 21). Overall, misclassifications were limited to 12 of 202 external samples, supporting consistent performance across the evaluated external cohorts.

Per-class performance metrics for MSI status classification are summarized in Supplementary Table S8. The model showed high precision, recall, and AUC values for both MSI-H and MSS classes across cohorts. In the external cohorts, MSI-H recall remained high (Set-2: 0.95; Set-3: 0.92), while precision ranged from 0.83 to 0.89. Similarly, the MSS class maintained a precision and recall above 0.94 across all cohorts. These findings indicate balanced performance for both MSI status classes in the evaluated cohorts.

**Fig. 4:**
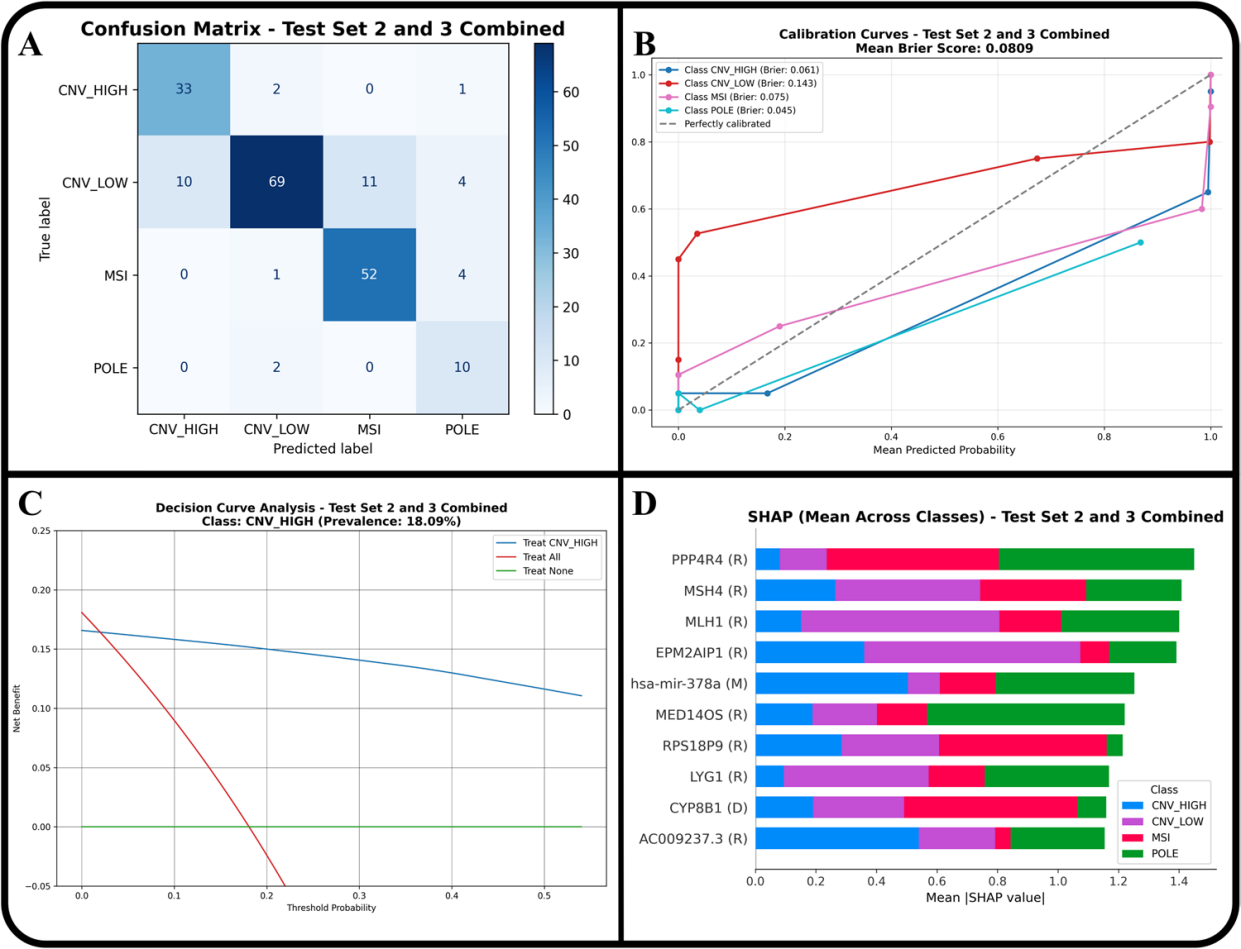
Performance and interpretability of the Multi-Layer Perceptron (MLP) model for genomic subtype classification on combined test sets. (**A**) Confusion matrix illustrating classification performance for the four genomic subtypes (CNV-High, CNV-Low, MSI, and POLE). (**B**) Calibration curves for each subtype showing agreement between predicted probabilities and observed outcomes. (**C**) Decision curve analysis demonstrating the net benefit of the model for CNV-High compared with treat-all and treat-none strategies. (**D**) SHAP summary plot highlighting the most important features and their impact on model predictions across the four classes

The MLP model for four-class genomic subtype classification showed consistent performance across cohorts, although confidence intervals were wider than for the binary MSI task, reflecting the increased difficulty of multi-class classification (Fig. 4A). On the internal validation set, the model achieved a balanced accuracy of 89.1%, AUC of 0.96, and macro F1-score of 87.2%. On Set-2, balanced accuracy was 86.2%, AUC was 0.96, and macro F1-score was 83.4%. On Set-3, the model achieved a balanced accuracy of 84.7%, AUC of 0.95, and macro F1-score of 75.5%. The wider confidence intervals on Set-3, particularly for macro F1-score, are consistent with smaller effective sample sizes per class and overlap between adjacent subtypes.

Per-class performance metrics for the genomic subtypes are summarized in Supplementary Table S9. Overall, the model demonstrated favorable performance across the major subtypes, with high precision, recall, and one-vs-rest AUC values for CNV-HIGH, CNV-LOW, and MSI across all cohorts. Despite being the least represented subtype, POLE maintained high recall (internal validation: 0.92; Set-2: 0.86; Set-3: 0.80) and strong one-vs-rest AUC values (0.89–0.97). However, precision declined in Set-3 (precision = 0.36), likely reflecting the small number of POLE cases (*n* = 5). Accordingly, POLE-specific estimates in the external cohorts (Set-2: *n* = 7; Set-3: *n* = 5) should be interpreted with caution and warrant validation in larger cohorts.

### Calibration curves

Calibration was assessed relative to a no-skill reference classifier that assigns the marginal class prevalence as the predicted probability for all samples. For the MSI model, the no-skill Brier score on the combined external set is 0.203 (MSI-H prevalence: 28.2%), compared to the model Brier score of 0.060, representing a substantial improvement over the no-skill baseline. For the genomic subtype model, the no-skill mean Brier score is 0.162 (computed per-class from subtype prevalences: CNV-High 18.1%, CNV-Low 33.3%, MSI 28.6%, POLE 8.0%), compared to the model mean Brier score of 0.081, again representing a substantial reduction relative to baseline. Calibration quality claims are therefore made relative to this no-skill reference rather than to perfect calibration alone. Regarding per-class calibration, POLE showed the lowest Brier score (0.045); however, as visible in Fig. 4-B, the POLE calibration curve is populated only up to a predicted probability of approximately 0.85, reflecting the low prevalence of this subtype and the model’s tendency to assign moderate rather than high-confidence POLE predictions. Claims of superior POLE calibration should therefore be interpreted within this limited probability range rather than across the full 0–1 spectrum. CNV-Low showed the greatest deviation from the diagonal (Brier score: 0.143), consistent with the known biological heterogeneity of this subtype.

ECE and MCE were computed for both top performing models across all evaluation sets. The LightGBM MSI classifier was well-calibrated without recalibration (validation ECE: 0.026; Set-2: 0.056; Set-3: 0.049; combined external: 0.049), consistent with the low Brier score of 0.060, and recalibration was not attempted. The MLP genomic subtype classifier showed moderate miscalibration on the validation set (ECE: 0.094), exceeding the pre-specified threshold of 0.05, and temperature scaling was applied (T = 4.344), fit on the internal validation set only. Post-recalibration ECE improved across all sets: validation (0.094 → 0.069), Set-2 (0.162 → 0.086), Set-3 (0.150 → 0.062), and combined external (0.143 → 0.052). Although temperature scaling improved overall calibration, residual instability remained for low-prevalence classes such as POLE. Brier score on the combined external set improved from 0.081 to 0.070. MCE on Set-3 did not improve after recalibration (0.553 → 0.613), reflecting residual worst-case bin instability on a small external cohort with low-prevalence subtypes rather than a systematic recalibration failure. Complete pre- and post-recalibration statistics are provided in Supplementary Table S10.

### Decision curve

Figure 3-C shows the decision curve analysis for the MSI-H versus MSS model (prevalence: 28.2%). The “treat MSI-H” strategy consistently provided higher net benefit than “treat-all” and “treat-none” across most threshold probabilities (0.05–0.75). The net benefit was observed at low-to-moderate thresholds (0.10–0.50), where the model clearly outperformed alternative strategies. Net benefit declined at higher thresholds but remained positive, indicating sustained usefulness under stricter decision criteria.

For Genomic subtype, decision curve analysis was performed to evaluate the net benefit of the model across predefined threshold probability ranges for each molecular subtype (Fig. 4-C). For the POLE subtype (threshold range: 0.05–0.30; prevalence: 6.03%), the model demonstrated a positive net benefit across the evaluated threshold range (Supplementary Fig. 3-A). The model-based strategy yielded higher net benefit than the treat-all and treat-none reference strategies. However, since ProMisE-based classification, MMR-IHC/MSI testing, POLE sequencing, p53-IHC, and integrated clinicopathological models were unavailable for comparison, these findings should be interpreted as exploratory assessment of model net benefit and not as evidence of clinical utility. In addition, given the low prevalence of POLE tumors and the small absolute net benefit, these findings should be interpreted cautiously and require validation in larger cohorts before clinical application. Net benefit gradually decreased at higher thresholds but remained positive up to 0.30. For CNV_HIGH (threshold: 0.20–0.50; prevalence: 18.09%), the model achieved a sustained positive net benefit throughout the selected range (Supplementary Fig. 3-B). The “treat CNV_HIGH” curve remained clearly above the “treat-all” and “treat-none” strategies, particularly between 0.25 and 0.45, suggesting optimal net benefit within this interval. In the case of MSI (threshold: 0.10–0.50; prevalence: 28.64%), the model provided substantial net benefit across most of the evaluated range. Between thresholds of approximately 0.15 and 0.45, the “treat MSI” strategy showed the highest net benefit, outperforming alternative strategies (Fig. 4-C). A decline was observed beyond 0.50, indicating reduced utility at very high confidence levels. For CNV_LOW (threshold: 0.15–0.60; prevalence: 47.24%), the model showed the strongest and most stable net benefit (Supplementary Fig. 3-C). The “treat CNV_LOW” curve remained well above both baseline strategies across the entire threshold range, with consistently high net benefit. Even at higher thresholds approaching 0.60, net benefit remained substantial.

### SHAP

SHAP analysis for the MSI model provides detailed insight into how individual molecular features contribute to the model’s predictions. Features are annotated by data type, where R denotes RNA-seq, D denotes DNA methylation, and M denotes miRNA expression. Features such as *MLH1*,* C3orf63*, and *PTMAP4* show the largest absolute SHAP values, indicating their dominant influence on MSI classification (Fig. 3-D). Each point represents a sample, with color reflecting feature expression level. High feature values (red) and low values (blue) are distributed asymmetrically along the SHAP axis, demonstrating that expression changes systematically shift predictions toward MSI-H or MSS. For example, elevated *MLH1* expression strongly drives predictions toward the MSS class, while reduced expression shifts the model toward the MSI-H class.

The stacked SHAP representation demonstrates that different features contribute differentially across subtypes, reflecting biologically distinct molecular signatures captured by the model (Fig. 4-D). *MLH1*,* MSH4*, and *PPP4R4*, emerged as key contributors for MSI and CNV_LOW classification. *EPM2AIP1* and *MED14OS* showed strong influence across multiple classes, indicating their role in general subtype discrimination. The microRNA *hsa-miR-378a* exhibited high importance, especially for CNV_HIGH and *POLE* predictions. *RPS18P9* and *CYP8B1* contributed predominantly to MSI and CNV_LOW predictions, while *AC009237.3* showed strong relevance for CNV_HIGH classification. For the *POLE* class (Supplementary Fig. 4-A), *CYP8B1* and *PPP4R4* were the most influential genes, while several microRNAs (*hsa-miR-139*,* hsa-miR-548j*,* hsa-miR-934*, and *hsa-miR-659*) also contributed substantially. In the CNV_HIGH class (Supplementary Fig. 4-B), CDKN2A emerged as the dominant predictor, followed by *AC009237.3* and *RPS27L*. The microRNA *hsa-miR-378a* showed strong influence. For the MSI class (Supplementary Fig. 4-C), mismatch repair–related genes, particularly *MLH1*,* EPM2AIP*1, and *MSH4*, were the strongest contributors. In the CNV_LOW class (Supplementary Fig. 4-D), *MED14OS* and *PPP4R4* were the most influential features, together with *hsa-miR-378a* and *CD8B2*. The SHAP scores for both the models can be found in Supplementary Table S11-S12.

### Survival analysis

Kaplan–Meier analysis showed broadly similar overall survival between MSI-H and MSS patients in the TCGA cohort (Supplementary Fig. 5A). In a multivariable Cox proportional hazards model adjusted for age at diagnosis, histological type, and MSI status, genomic subtype was entered as a categorical variable with POLE, the best-prognosis molecular subtype, as the reference group. Relative to POLE, CNV-High tumors showed significantly worse overall survival (HR = 14.5, 95% CI 1.90–110.95, *p* = 0.010), whereas CNV-Low (HR = 3.95, 95% CI 0.51–30.77, *p* = 0.19) and MSI (HR = 2.07, 95% CI 0.27–15.65, *p* = 0.48) tumors did not differ significantly from POLE. MSI-H status showed no independent association with overall survival (HR = 3.03, 95% CI 0.19–48.95, *p* = 0.44). The wide confidence intervals reflect the small number of POLE cases serving as the reference group. Complete multivariable results for all covariates are provided in Supplementary Table S13. Survival stratification based on model-selected molecular features is shown in Supplementary Fig. 5B–F. None of the individual features retained a statistically significant association with overall survival when entered together and adjusted for age, histological type, genomic subtype, and MSI status, indicating that the separation seen in the median-stratified Kaplan–Meier curves was largely accounted for by established clinical and molecular covariates.

Kaplan–Meier analysis demonstrated differences in overall survival across genomic subtypes (Supplementary Fig. 6A), consistent with established TCGA-defined prognostic patterns, with POLE tumors exhibiting the most favorable survival and CNV-High tumors the poorest. Survival stratification based on model-selected features is shown in Supplementary Fig. 6B–F. As in the MSI model, none of these features showed an independent prognostic association after mutual adjustment and adjustment for genomic subtype, MSI status, age, and histological type, supporting their role as molecular classifiers rather than independent prognostic biomarkers (Supplementary Table S14). Overall, these exploratory analyses indicate that EMMA-STRAT-selected features capture subtype-associated biological variation, while genomic subtype remains the principal determinant of prognosis.

### Downstream analysis

**Fig. 5:**
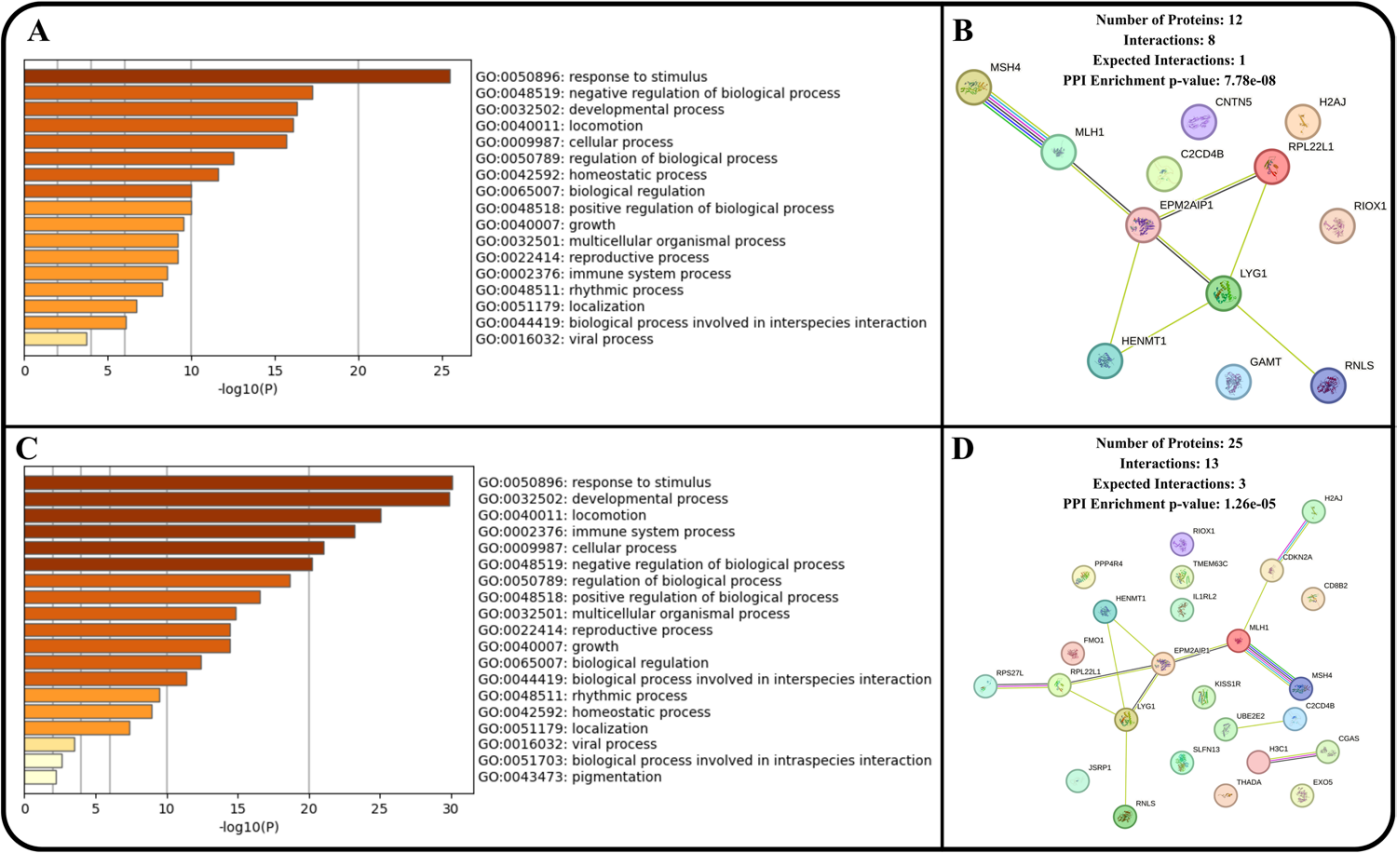
Functional enrichment and protein–protein interaction (PPI) network analysis of model-selected features. (**A**) Gene Ontology (GO) biological process enrichment analysis for validated target genes derived from the MSI model (SVM, 20 features). Bars represent –log10(P) values. (**B**) STRING protein–protein interaction network constructed from the MSI-derived protein set. Network statistics, including observed and expected interactions and PPI enrichment p-value, are shown. (**C**) Gene Ontology (GO) biological process enrichment analysis for validated target genes derived from the genomic subtype model (LASSO, 50 features). Bars represent –log10(P) values. (**D**) STRING protein–protein interaction network constructed from the genomic subtype–derived protein set (25 proteins). Network statistics, including observed and expected interactions and PPI enrichment p-value, are shown

To assess the biological relevance of the model-selected features, downstream functional and network analyses were performed on the RNA and miRNA features derived from the top-performing MSI and genomic subtype models. Functional enrichment analysis revealed that genes derived from the model-selected features were significantly enriched in broad biological processes, including response to stimulus, developmental process, regulation of biological process, cellular process, and immune system process (Fig. 5A and C). Additional enriched terms included homeostatic process, growth, locomotion, and viral process, suggesting involvement in both regulatory and stress-response pathways.

STRING network analysis demonstrated significant protein–protein interaction enrichment in both gene sets (Fig. 5B and D). For the MSI-derived targets, 12 proteins formed 8 interactions (expected interactions: 1; PPI enrichment *p* = 7.78 × 10⁻⁸), indicating a highly interconnected network beyond random expectation. For the genomic subtype model, 25 proteins formed 13 interactions (expected interactions: 3; PPI enrichment *p* = 1.26 × 10⁻⁵), similarly supporting functional connectivity. Notably, mismatch repair–related proteins such as *MLH1* and *MSH4* occupied central network positions, linking regulatory and immune-associated components. These findings suggest that the selected model features converge on biologically coherent pathways and form significantly enriched interaction networks, supporting their biological plausibility and functional relatedness in tumor biology.

Cross-omics methylation–RNA correlation analysis further supported the biological plausibility of the selected methylation features (Supplementary Table S15). A subset of model-selected methylation probes showed significant inverse associations with matched transcript levels, including several *HOX* family genes, consistent with methylation-associated transcriptional repression. *HOX* genes regulate developmental patterning and differentiation and are frequently dysregulated in cancer [78]. However, not all predictive methylation features showed corresponding cis transcript-level changes, indicating that some methylation signals may reflect broader subtype-associated epigenetic patterns rather than direct local gene repression. These findings partially address cross-omics interpretation, though comprehensive miRNA–target mRNA analyses and experimental validation remain necessary.

### Benchmarking against existing multi-omics frameworks

Comparison of EMMA-STRAT against three *Flexynesis* integration strategies is presented in Table 3. While *Flexynesis* methods achieved moderate internal validation performance, all three strategies showed substantial performance degradation on external cohorts, indicating limited cross-cohort generalisation. For MSI classification, *CrossModalPred* achieved the highest internal balanced accuracy among *Flexynesis* methods (86.6%) but dropped markedly on Set-2 (58.2%), recovering partially on Set-3 (87.5%). *DirectPred* and *supervised_vae* both reached 81.2% internally but fell to 56.4% and 54.4% respectively on Set-2, this performance approaching random chance for this binary task. In contrast, EMMA-STRAT maintained balanced accuracy of 98.1% internally, 94.9% on Set-2, and 93.1% on Set-3, with no meaningful degradation across cohorts. For genomic subtype classification, the pattern was more pronounced. *CrossModalPred* achieved 75.8% internally but declined to 61.9% on Set-2 and 54.7% on Set-3 which is a drop of 21.1% points from internal to Set-3. *supervised_vae* fell from 72.9% to 57.2% on Set-3. *DirectPred* showed the most severe degradation for MSI on Set-2 (56.4%). EMMA-STRAT achieved 89.1% internally and maintained 86.2% and 84.7% on Set-2 and Set-3 respectively, representing a maximal degradation of only 4.4% points across cohorts. These results demonstrate that *Flexynesis* strategies, while capable of fitting the training distribution, do not generalise reliably to independent CPTAC cohorts for this task. The systematic performance drop observed across all three *Flexynesis* methods on external sets underscores the importance of external validation.

**Table 3:** Benchmarking of EMMA-STRAT against Flexynesis integration strategies on external validation sets

MSI Status						
Method	Internal Validation (Set-1)		External Set-2		External Set-3	
	Accuracy	Macro F1	Accuracy	Macro F1	Accuracy	Macro F1
*Flexynesis — DirectPred*	81.2%	85.5%	56.4%	87.9%	80.6%	83.9%
*Flexynesis — Supervised VAE*	81.2%	85.5%	54.4%	81.6%	82.6%	85.1%
*Flexynesis — CrossModalPred*	86.6%	88.4%	58.2%	82.8%	87.5%	87.3%
EMMA-STRAT (LightGBM)	98.1%	98.1%	94.9%	92.7%	93.1%	92.8%

Genomic Subtype						
Method	Internal Validation (Set-1)		External Set-2		External Set-3	
	Accuracy	Macro F1	Accuracy	Macro F1	Accuracy	Macro F1
*Flexynesis — DirectPred*	69.3%	78.1%	68.5%	83.1%	65.2%	80.2%
*Flexynesis — Supervised VAE*	72.9%	80.2%	59.9%	74.2%	57.2%	73.2%
*Flexynesis — CrossModalPred*	75.8%	81.5%	61.9%	74.5%	54.7%	68.5%
EMMA-STRAT (MLP)	89.1%	87.2%	86.2%	83.4%	84.7%	75.5%

### Interactive results browser

To support transparency and facilitate exploration of the complete results, an interactive web-based results browser was developed and deployed via GitHub Pages at https://naisarg14.github.io/EMMA-STRAT-web-viewer/index.html. The browser comprises four pages: (i) a Model Results explorer providing filterable tabular views of balanced accuracy, macro F1-score, and AUC across all 1,152 trained model configurations, omics layers, feature selection methods, and evaluation sets; (ii) a Selected Features page listing the features chosen for the top-performing MSI and genomic subtype models with their omics layer annotations; (iii) a SHAP Analysis page displaying per-class feature importance scores for each model; and (iv) a *Flexynesis* Comparison page presenting side-by-side performance of EMMA-STRAT against the three *Flexynesis* integration strategies across all evaluation sets. All data is served as static JSON files. Screenshots of each page are provided in Supplementary Figure S7-S11.

## Discussion

EMMA-STRAT (Endometrial cancer Multi-omics Machine learning Architecture for STRATification) was developed as a supervised multi-omics framework for the classification of MSI status and molecular subtypes in uterine corpus endometrial carcinoma (UCEC). The framework integrates mRNA expression, miRNA expression, and DNA methylation data through standardized preprocessing and feature elimination to enhance prediction. EMMA-STRAT’s primary strength is its cross-cohort evaluation, demonstrated through external validation on two independent CPTAC cohorts, supporting its use as a research-oriented framework for molecular stratification in UCEC.

Single-omics benchmarking revealed a clear hierarchy (RNA > DNA methylation > miRNA), similar to previous studies [53]. Notably, compact sets of RNA features enabled strong MSI classification performance with as few as 10 features, indicating that the most discriminative signals for this task are highly concentrated in gene expression data. On the other hand, DNA methylation models required substantially larger feature sets to reach comparable accuracy, consistent with the more dispersed distribution of epigenetic changes across the genome. miRNA expression yielded the lowest standalone performance, likely due to its indirect regulatory effects and greater susceptibility to technical noise [54]. Nevertheless, miRNA-based models showed reasonable cross-cohort stability, suggesting that they capture regulatory information complementary to the other layers and supporting their inclusion in the multi-omics framework.

Integrating three omics layers improved model performance over the best single-omics approach, increasing balanced accuracy by 5–7.5% points for both tasks. Partial overlap in feature sets for MSI status and genomic subtype classification shows each omics layer provides shared and unique discriminative information. This in turn aligns with EC tumor biology where complex interactions among genomic, epigenomic, and post-transcriptional mechanisms are better captured by multi-omics integration than single-layer approaches [55, 56, 79].

Hyperparameter optimization using *Optuna* with cross-validation mitigated overfitting in high-dimensional, low-sample-size settings. The selected configurations maintained consistent performance across independent cohorts, suggesting that task-specific feature selection and model optimization contributed to stable cross-cohort performance. Analysis showed optimal strategies are task-dependent: SVM-based feature elimination with smaller feature sets suited MSI classification, while LASSO selection with larger sets preserved subtype-specific signals for multi-class discrimination [57]. Moreover, task-dependence of optimal feature selection parameters has practical implications for multi-omics machine learning pipelines, where such choices are treated as fixed hyperparameters rather than problem-dependent decisions, limiting performance and interpretability [58].

Direct benchmarking against *Flexynesis* further highlights the advantages of EMMA-STRAT’s design. In this implementation, *Flexynesis* strategies achieved moderate internal validation performance but showed lower external performance than EMMA-STRAT across the evaluated CPTAC cohorts. This comparison should be interpreted cautiously because EMMA-STRAT and *Flexynesis* employ different modeling strategies and native optimization procedures. Whereas *Flexynesis* is designed as a general-purpose multi-omics framework, EMMA-STRAT uses task-specific feature selection and model optimization tailored to the classification problem. Despite this specialization, EMMA-STRAT maintained consistent performance across independent external cohorts, suggesting that task-specific optimization may improve predictive performance and cross-cohort stability in the evaluated dataset.

The MSI classifier showed high sensitivity for MSI-H cases on external evaluation, correctly identifying 20 of 21 true MSI-H samples. This finding is relevant because MSI-H/MMR-deficient status is associated with Lynch syndrome screening and potential responsiveness to immune checkpoint inhibitor therapy [59]. The higher precision observed for MSS compared with MSI-H reflects underlying class imbalance, consistent with epidemiological distributions [60]. Importantly, the model maintained strong performance despite differences in MSI determination methodologies across datasets [21, 22, 61]. Compared with existing computational MSI detection approaches as benchmarked by Anthony and Seoighe (2024), this framework provides competitive performance on multi-omics expression data, though direct comparison is limited by differences in input data types and cohort composition [62]. However, EMMA-STRAT should not be interpreted as a replacement for established MSI/MMR testing or as a clinically deployable decision-making model at this stage. Rather, these results suggest that multi-omics molecular profiles can capture MSI-associated signals in research cohorts where such data are available. Prospective studies with complete ProMisE, MMR-IHC/MSI testing, POLE sequencing, p53-IHC, and clinicopathological annotations will be required to determine whether this approach adds value beyond established clinical workflows [63]. Model evaluation extended beyond accuracy to include calibration and net benefit.

Bootstrapped confidence interval analysis further substantiates the reliability of reported performance. For the LightGBM MSI classifier, the lower bounds of the 95% CIs for balanced accuracy on Set-2 and Set-3 remained well above random chance at 88.7% and 87.3% respectively, while the narrow AUC interval on Set-3 indicated stable and consistent discrimination despite methodological heterogeneity in MSI status determination across cohorts. For the MLP genomic subtype classifier, wider confidence intervals on Set-3 reflect the smaller per-class sample sizes and the greater inherent difficulty of four-class discrimination. Nonetheless, AUC lower bounds exceeding 0.87 across all external sets for both models confirm that classification performance is generalisable beyond the training cohort.

The MSI model demonstrated substantially better probabilistic calibration than a no-skill baseline, indicating reliable probability estimates across the evaluated range. For genomic subtyping, calibration varied across classes, with *POLE* and CNV-High showing lower Brier scores, whereas CNV-Low exhibited greater deviation, likely reflecting its biological heterogeneity [64]. Decision curve analysis showed higher net benefit than treat-all and treat-none strategies across selected threshold probabilities, but these findings should be interpreted as exploratory because direct comparison with *ProMisE*-based or integrated clinicopathological decision strategies was not performed [65, 66].

SHAP analysis provided detailed insight into feature contributions and confirmed the biological relevance of the models [67]. MLH1 emerged as the dominant predictor for MSI classification, with contribution direction consistent with known biology [68]. Across genomic subtypes, distinct molecular features were identified, including mismatch repair genes (*MLH1*, *MSH4*), copy-number–associated features (*CDKN2A*), and epigenetic regulators such as *MED14OS*. Several additional features identified by the model have established roles in cancer-related pathways, including *PPP4R4*, *EPM2AIP1*, *PTMAP4*, and *hsa-miR-378a* [69–72]. Conversely, *CYP8B1* has been reported to exhibit tumor-suppressive effects in certain cancer contexts [73]. Notably, *TASOR* (*C3orf63*) methylation emerged as a significant MSI-associated feature [74]. As a component of the HUSH complex involved in epigenetic silencing, its role in endometrial cancer remains underexplored, suggesting a potential link between epigenetic regulation and mismatch repair pathways. Importantly, the models preferentially highlighted subtype-discriminative genes rather than general tumor biomarkers.

Protein–protein interaction analysis demonstrated significant enrichment, indicating that the selected features form biologically coherent networks rather than random associations [75, 76]. Cross-omics methylation-RNA correlation analysis further supported this coherence: among model-selected methylation features with detectable RNA expression, 27 of 52 testable genes exhibited significant inverse correlations between M-values and transcript abundance (FDR q < 0.05), consistent with methylation-associated transcriptional silencing. The recurrence of HOX family members (HOXA4, HOXA7, HOXA10, and HOXB13) as silencing-consistent features across both models is in agreement with previous reports of HOX cluster hypermethylation in endometrial cancer, supporting the biological relevance of the epigenetic information captured by the framework.

Survival analyses were performed as an exploratory secondary analysis to assess the biological plausibility of model-selected features. Multivariable Cox regression identified genomic subtype as the dominant prognostic determinant, with CNV-High tumors showing markedly worse overall survival than POLE tumors (HR = 14.5, *p* = 0.010), consistent with established TCGA-defined prognostic patterns in UCEC [77, 80, 81]. Confidence intervals were wide owing to the small number of POLE cases in the reference group, so these estimates should be read as directionally consistent with known subtype prognosis rather than as precise effect sizes. In contrast, individual model-selected features did not retain statistically significant associations after adjustment for major clinical and molecular confounders, suggesting that their prognostic relevance is largely mediated through subtype membership. These findings indicate that EMMA-STRAT-selected features primarily function as molecular classifiers rather than independent prognostic biomarkers, and further validation in larger prospective cohorts will be required to establish any independent prognostic utility.

All model results are openly accessible through an interactive web browser that enables exploration of performance metrics across 1,152 model configurations, providing access to feature lists, SHAP importance scores, and *Flexynesis* benchmarking comparisons for verification and reuse, supporting transparency and reproducibility.

This study has several limitations. EMMA-STRAT was developed and evaluated retrospectively using publicly available TCGA and CPTAC multi-omics cohorts. Although Set-2 and Set-3 served as independent external validation cohorts, prospective multicenter validation in clinically collected samples and experimental validation of selected molecular features remain necessary. The framework did not incorporate standard clinicopathological variables, including stage, grade, histological subtype, treatment history, age, p53 status, mismatch repair immunohistochemistry, or POLE sequencing results. Consequently, this study does not establish incremental clinical value over ProMisE or integrated clinicopathological risk models, and direct comparisons with these established frameworks are needed. In addition, EMMA-STRAT requires mRNA, miRNA, and DNA methylation data, which are not routinely available in many clinical settings. The associated cost, tissue requirements, and bioinformatics infrastructure currently limit immediate clinical implementation, and the framework should therefore be viewed as a research-oriented molecular stratification approach rather than a replacement for established PCR, immunohistochemistry, or sequencing-based assays. Accordingly, EMMA-STRAT should not be interpreted as a clinically deployable decision-making model at this stage, and this study does not demonstrate added value beyond ProMisE, MMR-IHC/MSI testing, POLE sequencing, p53-IHC, or integrated clinicopathological risk models. Furthermore, the MSI classifier was developed solely for the binary distinction between MSI-H and MSS tumors, as no MSI-L cases were present in the available cohorts. It cannot classify MSI-L tumors, and its behavior on such cases in future cohorts is undetermined; MSI-L samples would require separate model development and validation. Although three omics layers were integrated for prediction and methylation–RNA correlation analysis was added for selected methylation features, cross-omics regulatory interpretation remains incomplete. In particular, systematic miRNA–target mRNA analyses and experimental validation were not performed, limiting downstream biological interpretation. While consistent performance across independent external cohorts mitigates this concern, the limited number of POLE cases restricts the reliability of POLE-specific performance, calibration, and decision-curve estimates. Finally, calibration and survival analyses should be interpreted cautiously. Calibration analyses were descriptive, and survival analyses were exploratory; therefore, model-selected features should not be regarded as independently validated prognostic biomarkers. Future studies should evaluate EMMA-STRAT in larger clinically annotated cohorts with standardized treatment information, prospective follow-up, experimental validation, and direct comparison with established molecular and clinicopathological risk stratification systems.

## Conclusion

This study presents EMMA-STRAT, a supervised multi-omics machine learning framework for MSI status prediction and molecular subtype classification in endometrial carcinoma. By integrating mRNA expression, miRNA expression, and DNA methylation data, the framework supported prediction of MSI-H versus MSS status and TCGA-defined genomic subtypes across internal validation and two independent external cohorts. SHAP analysis, functional enrichment, protein–protein interaction analysis, and methylation–RNA correlation analysis supported the biological plausibility of selected molecular features. To facilitate transparency and reproducibility, model outputs, feature importance scores, and benchmarking comparisons are available through an interactive results browser https://naisarg14.github.io/EMMA-STRAT-web-viewer/index.html. EMMA-STRAT should currently be viewed as a research-oriented molecular stratification framework rather than a clinically deployable decision-making model. Prospective validation, incorporation of clinicopathological variables, and direct comparison with established approaches including ProMisE, MMR-IHC/MSI testing, POLE sequencing, p53-IHC, and integrated clinicopathological risk models, will be required before clinical implementation can be considered.

## Supplementary Information

Below is the link to the electronic supplementary material.

Supplementary Material 1: Supplementary Table S1. Integrated summary of multi-omics datasets across each omics layer. Supplementary Table S2. Features selected for MSI status classification. Supplementary Table S3. Features selected for genomic subtype classification. Supplementary Table S4. Performance of single-omics models for MSI status classification across all evaluation sets. Supplementary Table S5. Performance of single-omics models for genomic subtype classification across all evaluation sets. Supplementary Table S6. Performance of multi-omics models for MSI status classification across all evaluation sets. Supplementary Table S7. Performance of multi-omics models for genomic subtype classification across all evaluation sets. Supplementary Table S8. Performance metrics of the LightGBM model using 20 RFE-SVM-selected features for MSI status prediction across Validation set and External Validation Sets (Set-2 and Set-3). Supplementary Table S9. Performance metrics of the MLP model with RFE-LASSO-selected 50 features for genomic subtype (IC) classification across Validation set and External Validation Sets (Set-2 and Set-3). Supplementary Table S10. Calibration performance of the final MSI and integrated-classification (IC) models before and after temperature scaling. ECE, expected calibration error; MCE, maximum calibration error. Supplementary Table S1(1) SHAP feature importance scores for the MSI status classification model. Supplementary Table S1(2) Class-specific and mean SHAP feature importance scores for the genomic subtype classification model. Supplementary Table S1(3) Multivariable Cox proportional hazards model for overall survival in the TCGA cohort. Supplementary Table S1(4) Results of multivariable Cox proportional hazards analyses for the MSI and genomic subtype models. Supplementary Table S1(5) Cross-omics methylation-RNA Spearman correlation analysis for model-selected DNA methylation features. Supplementary Table S1(6) Software tools, packages, and databases used in this study.

Supplementary Material 2

Supplementary Material 3: Supplementary Fig. 1. Overlap of selected features between genomic subtype and MSI models. Supplementary Fig. 2. Principal component analysis (PCA) plots showing the distribution of samples from Set-1, Set-2, and Set-3 before and after batch correction. Supplementary Fig. 3. Decision curve analysis for genomic subtype classification across molecular subtypes on combined external test sets (Set-2 and Set-3). Supplementary Fig. 4. SHAP-based feature importance analysis for the genomic subtype model on the combined external test sets (Set-2 and Set-3). Supplementary Fig. 5. Survival analysis for MSI-H versus MSS classification and selected molecular features. Supplementary Fig. 6. Survival analysis by genomic subtype and selected molecular features. Supplementary Fig. 7. Screenshot of the EMMA-STRAT interactive results browser home page. Supplementary Fig. 8. Screenshot of the Model Results page of the EMMA-STRAT interactive results browser. Supplementary Fig. 9. Screenshot of the Selected Features page of the EMMA-STRAT interactive results browser. Supplementary Fig. 10. Screenshot of the SHAP Analysis page of the EMMA-STRAT interactive results browser. Supplementary Fig. 11. Screenshot of the Flexynesis Comparison page of the EMMA-STRAT interactive results browser.

## Data Availability

The mRNA expression, miRNA expression, and DNA methylation data used in this study are publicly available and were obtained from The Cancer Genome Atlas (TCGA) and the Clinical Proteomic Tumor Analysis Consortium (CPTAC) through the Genomic Data Commons (GDC) Data Portal (https://portal.gdc.cancer.gov; Data Release 44.0) for the Uterine Corpus Endometrial Carcinoma (UCEC) cancer type. Sample metadata for TCGA were retrieved from cBioPortal (https://www.cbioportal.org), and metadata for CPTAC cohorts were obtained from the LinkedOmics database (http://www.linkedomics.org). No new clinical data were generated in this study.

## Code availability

All code used for this study is publicly available in the project GitHub repository at https://github.com/naisarg14/EMMA-STRAT under the GNU General Public License v3 (GPL 3.0). An interactive results browser is accessible at https://naisarg14.github.io/EMMA-STRAT-web-viewer/index.html, with the corresponding repository available at https://github.com/naisarg14/EMMA-STRAT-web-viewer. Software tools, packages, and version numbers are listed in Supplementary Table S16.
