# Supplementary material for: EMMA-STRAT: a multi-omics based machine learning framework for stratification of endometrial carcinoma molecular subtypes and MSI status
Source: BioData Min. 2026 Jul 23;19:63. doi: 10.1186/s13040-026-00587-5 (PMC13474893; doi:10.1186/s13040-026-00587-5)
Supplement: Supplementary file 1 [file 13040_2026_587_MOESM2_ESM.pdf]

Supplementary Methods for "EMMA-STRAT: A Multi-Omics Based Machine Learning Framework for Stratification of Endometrial Carcinoma Molecular Subtypes and MSI Status"

Naisarg Patel[1,2], Andres Salumets[1,2,3*], Vijayachitra Modhukur [1,2*]

1. *Celvia CC AS, Tartu, Estonia*
2. *Department of Obstetrics and Gynecology, Institute of Clinical Medicine, University of Tartu, L. Puusepa 8, Tartu, Estonia*
3. *Division of Obstetrics and Gynecology, Department of Clinical Science, Intervention and Technology (CLINTEC), Karolinska Institute, and Karolinska University Hospital, Stockholm, Sweden*

*** To whom correspondence should be addressed.**
Dr. Vijayachitra Modhukur, Department of Obstetrics and Gynaecology, Institute of Clinical Medicine, University of Tartu, Tartu, Estonia. E-mail: vijayachitra.modhukur@ut.ee

*** Correspondence may also be addressed to**
Prof. Andres Salumets, Division of Obstetrics and Gynaecology, Department of Clinical Science, Intervention and Technology (CLINTEC), Karolinska Institutet, and Karolinska University Hospital, Stockholm, Sweden. E-mail: andres.salumets@ki.se

S1. Model construction and training details

All models were trained on feature matrices produced by the project's preprocessing pipeline and loaded via model_helper.get_sets(...), which returns (i) selected and scaled feature matrices for training and evaluation splits and (ii) encoded class labels. Hyperparameters were tuned with Optuna to maximise 5-fold stratified cross-validated macro-F1 on the training split (StratifiedKFold, n_splits=5, shuffle=True, random_state=19). Random seeds were fixed (NumPy seed = 19; TensorFlow seed = 19 for neural models). Class imbalance was handled using balanced class weights (either via model class_weight='balanced' or explicit per-class weights).

S1.1 k-Nearest Neighbours (KNN)

For the KNN classifier, each sample is represented by its scaled molecular feature vector and classification is performed by majority vote (or distance-weighted vote) among the k nearest samples in feature space using sklearn.neighbors.KNeighborsClassifier [1, 2].

Hyperparameters were optimised with Optuna over: number of neighbours k in [3, 50]; neighbour weighting scheme in {uniform, distance}; distance metric in {euclidean, manhattan, minkowski, chebyshev}; Minkowski exponent p in [1, 5] only when metric='minkowski' (otherwise p=2); and leaf_size in [10, 50].

The objective function computed the mean macro-F1 across 5-fold stratified cross-validation on the training split. The final KNN model was then refit on the full training split with the best hyperparameters and evaluated on the internal validation set and external test sets.

S1.2 LightGBM gradient-boosted decision trees (LGBM)

For the LightGBM classifier, boosted decision trees were trained using lightgbm.LGBMClassifier with balanced class weights (class_weight='balanced'), random_state=19, and multithreading enabled (n_jobs=-1). The objective function was selected based on the number of classes: objective='binary' for two-class problems and objective='multiclass' with num_class set accordingly for multi-class problems [3].

Hyperparameters were optimised with Optuna over: n_estimators in [100, 1000] (step 100); learning_rate in [0.01, 0.3] on a log scale; num_leaves in [15, 255]; max_depth in [3, 15]; min_child_samples in [5, 100]; subsample in [0.5, 1.0]; colsample_bytree in [0.5, 1.0]; L1 and L2 regularisation (reg_alpha, reg_lambda) each in [1e-8, 10] on a log scale; min_split_gain in [0.0, 1.0]; and subsample_freq in [1, 10] only when subsample < 1.0 (otherwise subsample_freq=0).

During cross-validation, each fold was trained with early stopping (stopping_rounds=50) using the fold's held-out partition as an evaluation set, and macro-F1 was computed on fold predictions. The final model was refit on the full training split using the best hyperparameters and evaluated on the internal validation set and external test sets.

Note: in the current lgbm.py script, Optuna is invoked with n_trials=1; the search space and objective function are as described above.

S1.3 Random Forest (RF)

For the Random Forest classifier, an ensemble of decision trees was trained using sklearn.ensemble.RandomForestClassifier with class_weight='balanced', random_state=19, and parallel training enabled (n_jobs=-1). Each tree is trained on a bootstrap sample (optional) and split criteria are chosen to maximise impurity reduction, with additional regularisation via minimum impurity decrease [4].

Hyperparameters were optimised with Optuna over: n_estimators in [50, 500] (step 50); max_depth in [3, 30]; min_samples_split in [2, 20]; min_samples_leaf in [1, 10]; max_features in {sqrt, log2, None}; bootstrap in {True, False}; min_impurity_decrease in [0.0, 0.2]; and max_samples in [0.5, 1.0] when bootstrap=True (otherwise max_samples=None).

Performance was assessed as mean macro-F1 across 5-fold stratified cross-validation on the training split. The final RF model was then trained on the full training split using the best hyperparameters and evaluated on the internal validation set and external test sets.

S1.4 Multilayer Perceptron (MLP)

For the MLP classifier, a fully connected neural network was implemented in TensorFlow/Keras as a feed-forward multilayer perceptron operating on scaled feature vectors. The network consists of an input layer followed by 2–4 hidden layers. Each hidden layer applies: Dense(hidden_dim, ReLU) with L2 weight regularisation; LayerNormalization; and Dropout. The output layer is Dense(num_classes) with no activation, producing class logits [5].

Hyperparameters were optimised with Optuna over: number of hidden layers in [2, 4]; hidden_dim in [64, 512] (step 64) and repeated across layers; dropout rate in [0.1, 0.5]; learning rate in [1e-4, 1e-2] on a log scale; batch_size in {32, 64, 128, 256}; and L2 regularisation strength l2_reg in [1e-6, 1e-3] on a log scale.

Training used the Adam optimiser with gradient clipping (clipnorm=1.0) and sparse categorical cross-entropy loss (from_logits=True). Class imbalance was handled via per-class weights computed from the training labels using sklearn.utils.class_weight.compute_class_weight('balanced'). Early stopping was applied based on a custom validation macro-F1 metric computed at the end of each epoch (patience=10 during hyperparameter optimisation; patience=20 during final training), restoring the best weights. During optimisation, each Optuna trial evaluated the mean validation macro-F1 across 5-fold stratified CV (up to 50 epochs per fold). The final model was trained on the full training split with the selected hyperparameters (up to 150 epochs with early stopping) and evaluated on the internal validation set and external test sets.

S1.5 Graph Neural Network (GNN)

For the GNN classifier, samples were represented as nodes in a sample-level graph (one node per patient), with each node feature vector comprising the scaled molecular features. A k-nearest neighbour (k-NN) graph was constructed in feature space using sklearn.neighbors.kneighbors_graph with mode='distance' (and include_self=True in the current implementation). For a chosen k, directed edges are formed between nodes according to the k-NN adjacency; raw Euclidean distances d_ij were transformed into similarity-based edge weights using a Gaussian (RBF) kernel:

$$w_{ij} = exp\left(-\frac{d_{ij}^2}{2\sigma^2}\right)$$

where d_{ij} is the Euclidean distance between nodes i and j in the molecular feature space, and σ is set adaptively as the mean of all k-NN distances in the graph, ensuring edge weights lie in the interval (0,1](0, 1] (0,1] with geometrically proximate samples receiving stronger connections. The resulting weighted adjacency was stored as an edge feature named 'weight' [6].

The GNN was implemented with TensorFlow GNN. Node features were first projected to a hidden dimension using a Dense(hidden_dim, ReLU) layer with L2 regularisation followed by Dropout. Message passing then proceeded through num_layers stacked mt_albis.MtAlbisGraphUpdate layers configured with: units=hidden_dim; message_dim=max(16, hidden_dim//2); attention_type='none'; simple_conv_reduce_type='sum' (to incorporate weighted aggregation); normalization_type='layer'; next_state_type='residual' (residual updates); and state_dropout_rate=dropout. After the final message-passing layer, node embeddings were read out directly (node classification setting) and mapped to class logits via a final Dense(num_classes) layer.

Hyperparameters were optimised with Optuna over: num_layers in [2, 4]; hidden_dim in [64, 512] (step 64); dropout rate in [0.1, 0.5]; learning rate in [1e-4, 1e-2] on a log scale; L2 regularisation l2_reg in [1e-6, 1e-3] on a log scale; and k (k_neighbors) in [5, 20].

Training used Adam with gradient clipping (clipnorm=1.0) and sparse categorical cross-entropy (from_logits=True). Validation macro-F1 was computed each epoch by applying softmax to logits and comparing argmax predictions to node labels; early stopping restored the best weights (patience=10 during hyperparameter optimisation; patience=20 for final training). Class imbalance was handled with balanced class weights computed from labels.

To avoid cross-split graph leakage, graphs were constructed independently from the feature matrices of each split (training fold, validation fold, internal validation set, and external test sets) rather than building a single transductive graph spanning multiple splits.

S1.6 Support Vector Machine (SVM)

For the SVM classifier, a kernel SVM was trained using sklearn.svm.SVC with probability estimates enabled (probability=True) and class imbalance handled via class_weight='balanced'. Input samples were represented by their scaled feature vectors [7].

Hyperparameters were optimised with Optuna over: C in [1e-2, 1e2] on a log scale; kernel in {rbf, linear, poly, sigmoid}; and gamma chosen either categorically as {'scale', 'auto'} or as a numeric value gamma_value in [1e-4, 1e1] on a log scale. For the polynomial kernel, degree was tuned in [2, 5] and coef0 in [0, 10]; for the sigmoid kernel, coef0 was tuned in [0, 10].

Performance was assessed as mean macro-F1 over 5-fold stratified cross-validation on the training split. The final SVM model was then refit on the full training split using the best hyperparameters and evaluated on the internal validation set and external test sets.

S2 Survival Analysis

Survival analyses were restricted to the TCGA cohort (Set-1), which provided sufficient follow-up duration and event counts for survival modelling; Sets 2 and 3 had insufficient follow-up duration and event counts for reliable Cox regression.

Kaplan–Meier survival curves were estimated using the kaplan_meier_estimator function from scikit-survival. Group-level curves were generated for MSI status (MSI-H vs MSS) and for genomic subtype (POLE, MSI, CNV-Low, CNV-High), and differences across these groups were assessed using the log-rank test via the compare_survival function from scikit-survival. For each model-selected molecular feature, additional Kaplan–Meier curves were generated by stratifying samples into high- and low-expression groups at the median value of that feature; these per-feature curves are presented for visualization only, with the annotated hazard ratio taken from the multivariable Cox model rather than from a log-rank test. Survival probability was plotted against follow-up time.

Multivariable Cox proportional hazards regression was performed using the CoxPHFitter function from the lifelines library to estimate adjusted hazard ratios (HRs) with 95% confidence intervals (CIs) and two-sided p-values. Two adjusted Cox models were constructed, First, a group-level multivariable model was fitted to assess the independent prognostic associations of MSI status and genomic subtype simultaneously. It included MSI status (MSI-H vs MSS, binary), age at diagnosis (continuous), histological type (endometrioid vs non-endometrioid, binary), and genomic subtype, modeled as a categorical variable with POLE, the established best-prognosis subtype, as the reference level. Hazard ratios for the MSI, CNV-Low, and CNV-High subtypes are therefore reported relative to POLE. Second, to assess the prognostic associations of the model-selected molecular features, a single multivariable model was fitted that entered all top-ranked features simultaneously, together with MSI status, age at diagnosis, histological type, and genomic subtype; the HR reported for each feature therefore reflects its association with overall survival mutually adjusted for the remaining features and the clinical covariates. All continuous covariates were included on their original scale without transformation. Sentinel missing values were coerced to NaN prior to fitting and complete-case analysis was applied.

The proportional hazards assumption was evaluated for every covariate in all three models using the Grambsch–Therneau test on scaled Schoenfeld residuals with rank-transformed time, implemented via the proportional_hazard_test function from lifelines.statistics. All covariates satisfied the proportional hazards assumption (all $p > 0.05$), consistent with the validity of the fitted Cox models. All survival findings are considered exploratory and hypothesis-generating, secondary to the primary classification task of EMMA-STRAT.

S3 Flexynesis Benchmark Design and Configuration

S3.1 Benchmark fairness rationale

EMMA-STRAT and Flexynesis represent two distinct design philosophies for supervised multi-omics learning. EMMA-STRAT employs an externalized optimization strategy, in which the feature selection method, feature subset size, classifier, and hyperparameters are treated as explicit components and independently benchmarked. In contrast, Flexynesis incorporates these processes internally through its native feature selection and automated hyperparameter optimization (HPO) procedures. Consequently,

a fair comparison requires that each framework be evaluated using its intended optimization strategy while maintaining identical input data, sample partitions, prediction targets, and evaluation procedures.

Accordingly, both frameworks were trained using the same post-quality-control and normalized mRNA, miRNA, and DNA methylation matrices. Identical sample partitions were used, consisting of the Set-1 training and internal validation cohorts and the external validation cohorts Set-2 and Set-3. The prediction tasks (four-class genomic subtype classification and binary MSI status classification), performance metrics, and evaluation scripts were also kept identical. Differences were limited to the feature selection and hyperparameter optimization stages. EMMA-STRAT employed task-specific feature selection approaches (ANOVA, RFE-SVM, RFE-RF, and RFE-LASSO) together with external Optuna-based hyperparameter optimization, whereas Flexynesis used its native variance filtering and Laplacian-score-based feature selection combined with the framework's built-in automated HPO. These components were intentionally not standardized across frameworks because they are integral to the respective methodologies. Imposing the feature selection or optimization strategy of one framework on the other would alter its intended operation and therefore would not constitute a fair comparison.

S3.2 Flexynesis configuration

Flexynesis (v1.1.8) was evaluated using all three supervised integration architectures available within the framework, namely DirectPred, supervised_vae, and CrossModalPred. A uniform configuration was applied across all architectures. Both classification endpoints, Genomic_Subtype and MSI_Status, were specified as categorical targets and modeled simultaneously in a single multi-task learning framework. All three omics modalities (mRNA, miRNA, and DNA methylation) were jointly supplied as input.

The native feature selection procedure implemented in Flexynesis was enabled. Following variance and missing-value filtering, Laplacian-score-based feature selection reduced the dimensionality from 27,366 to 5,415 features for mRNA, from 503 to 493 features for miRNA, and from 2,576 to 508 features for DNA methylation. Automated hyperparameter optimization was performed using 100 iterations (`hpo_iter = 100`) with a maximum of 10 training epochs (`epochs = 10`) and early stopping. The default Flexynesis search space was used, encompassing latent dimensionality, network architecture depth and width, learning rate, and regularization parameters.

Feature scaling was performed independently for each modality using StandardScaler, which was fitted exclusively on the Set-1 training partition and subsequently applied unchanged to the internal validation and external datasets, thereby preventing data leakage. Hyperparameter optimization and early stopping were conducted solely using the Set-1 training partition, while the external cohorts (Set-2 and Set-3) were reserved exclusively for independent evaluation and were not accessed during model fitting or optimization.

S3.3 Evaluation procedure

Predictions from all three Flexynesis architectures were scored using the same evaluation script as EMMA-STRAT. For internal validation, only the held-out Set-1 30% split was scored. Samples with missing or Unknown labels were excluded identically to the EMMA-STRAT evaluation. Primary metrics were balanced accuracy and macro-averaged F1-score. For multi-class AUROC, named-class probabilities were renormalised to sum to one per sample prior to one-vs-rest macro-averaged scoring.
