# Supplementary material for: EMMA-STRAT: a multi-omics based machine learning framework for stratification of endometrial carcinoma molecular subtypes and MSI status
Source: BioData Min. 2026 Jul 23;19:63. doi: 10.1186/s13040-026-00587-5 (PMC13474893; doi:10.1186/s13040-026-00587-5)
Supplement: Supplementary file 2 [file 13040_2026_587_MOESM3_ESM.pdf]

Supplementary Figures for "EMMA-STRAT: A Multi-Omics Based Machine Learning Framework for Stratification of Endometrial Carcinoma Molecular Subtypes and MSI Status"

Naisarg Patel[1,2], Andres Salumets[1,2,3*], Vijayachitra Modhukur [1,2*]

1. *Celvia CC AS, Tartu, Estonia*
2. *Department of Obstetrics and Gynecology, Institute of Clinical Medicine, University of Tartu, L. Puusepa 8, Tartu, Estonia*
3. *Division of Obstetrics and Gynecology, Department of Clinical Science, Intervention and Technology (CLINTEC), Karolinska Institute, and Karolinska University Hospital, Stockholm, Sweden*

*** To whom correspondence should be addressed.**
Dr. Vijayachitra Modhukur, Department of Obstetrics and Gynaecology, Institute of Clinical Medicine, University of Tartu, Tartu, Estonia. E-mail: vijayachitra.modhukur@ut.ee

*** Correspondence may also be addressed to**
Prof. Andres Salumets, Division of Obstetrics and Gynaecology, Department of Clinical Science, Intervention and Technology (CLINTEC), Karolinska Institutet, and Karolinska University Hospital, Stockholm, Sweden. E-mail: andres.salumets@ki.se

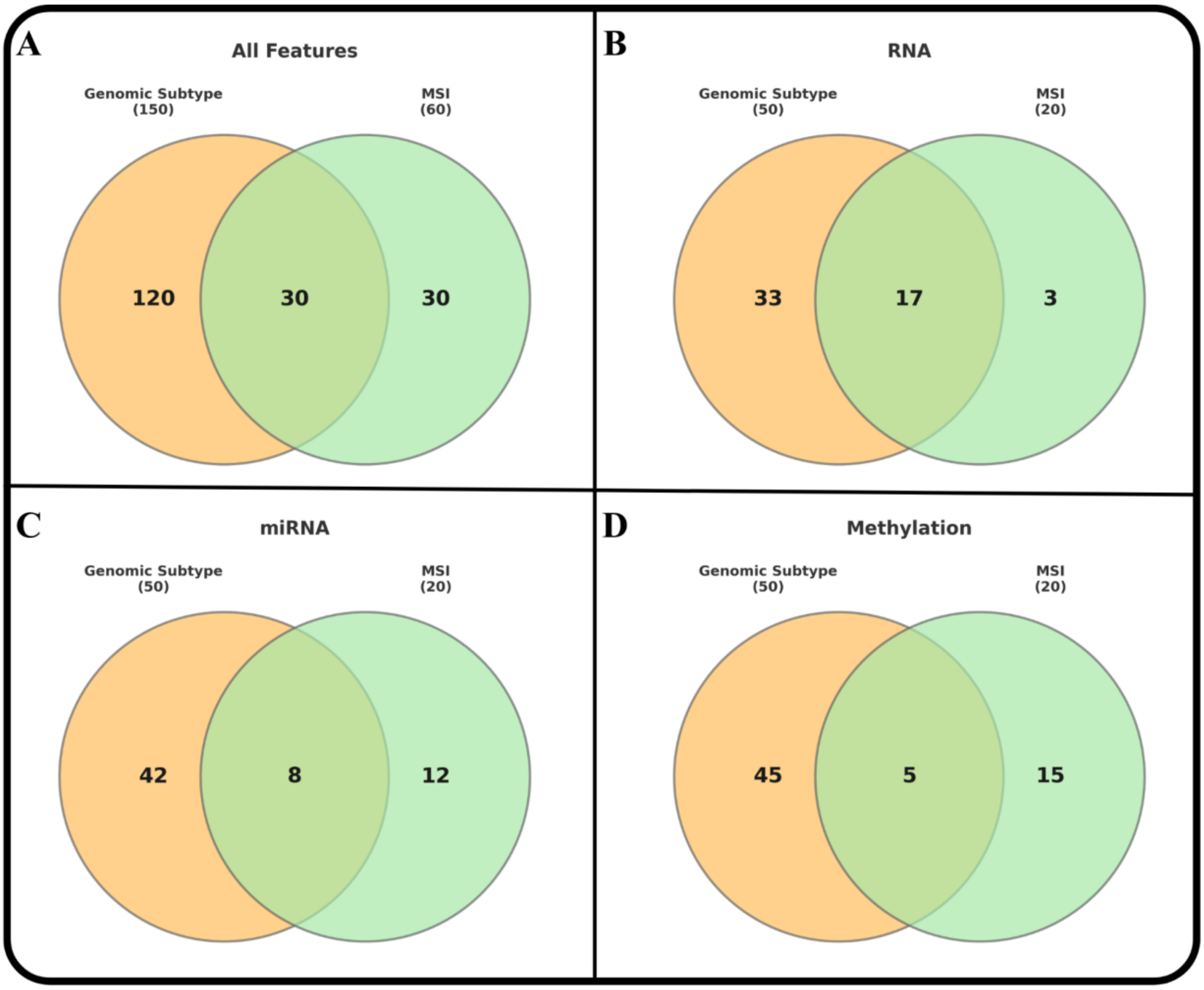

Supplementary Figure 1: Overlap of selected features between genomic subtype and MSI models. *Venn diagrams showing shared and unique features between the genomic subtype model and MSI model for (A) all features, (B) RNA features, (C) miRNA features, and (D) DNA methylation features. Numbers indicate feature counts in each category and their intersection.*

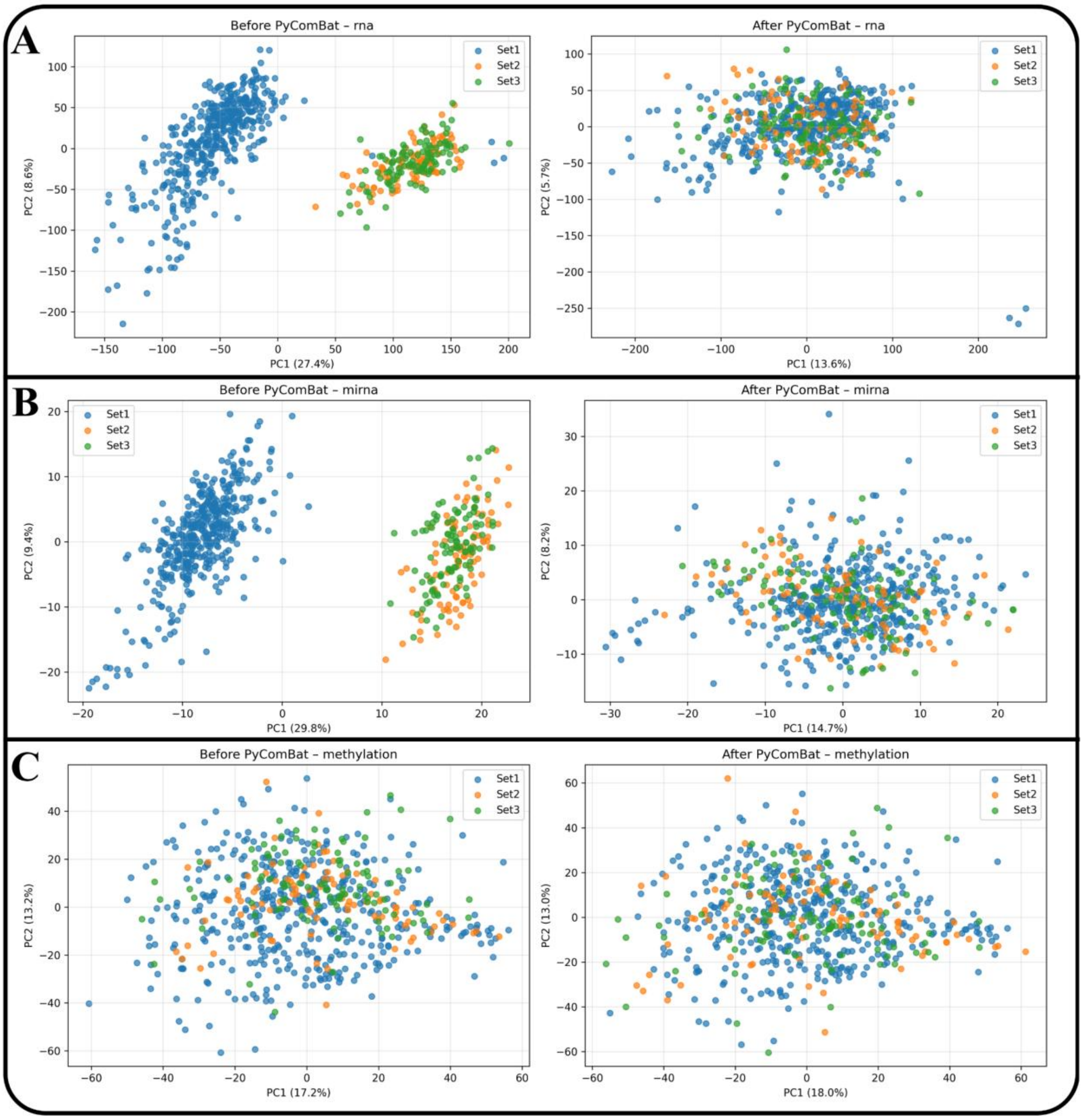

Supplementary Figure 2. Principal component analysis (PCA) plots showing the distribution of samples from Set-1, Set-2, and Set-3 before and after batch correction. (A) mRNA expression, (B) miRNA expression, and (C) DNA methylation data. The left panels show the first two principal components prior to batch correction, demonstrating pronounced batch-associated clustering for mRNA and miRNA data. The right panels show the corresponding distributions after batch correction. Percentages on the axes denote the proportion of variance explained by each principal component.

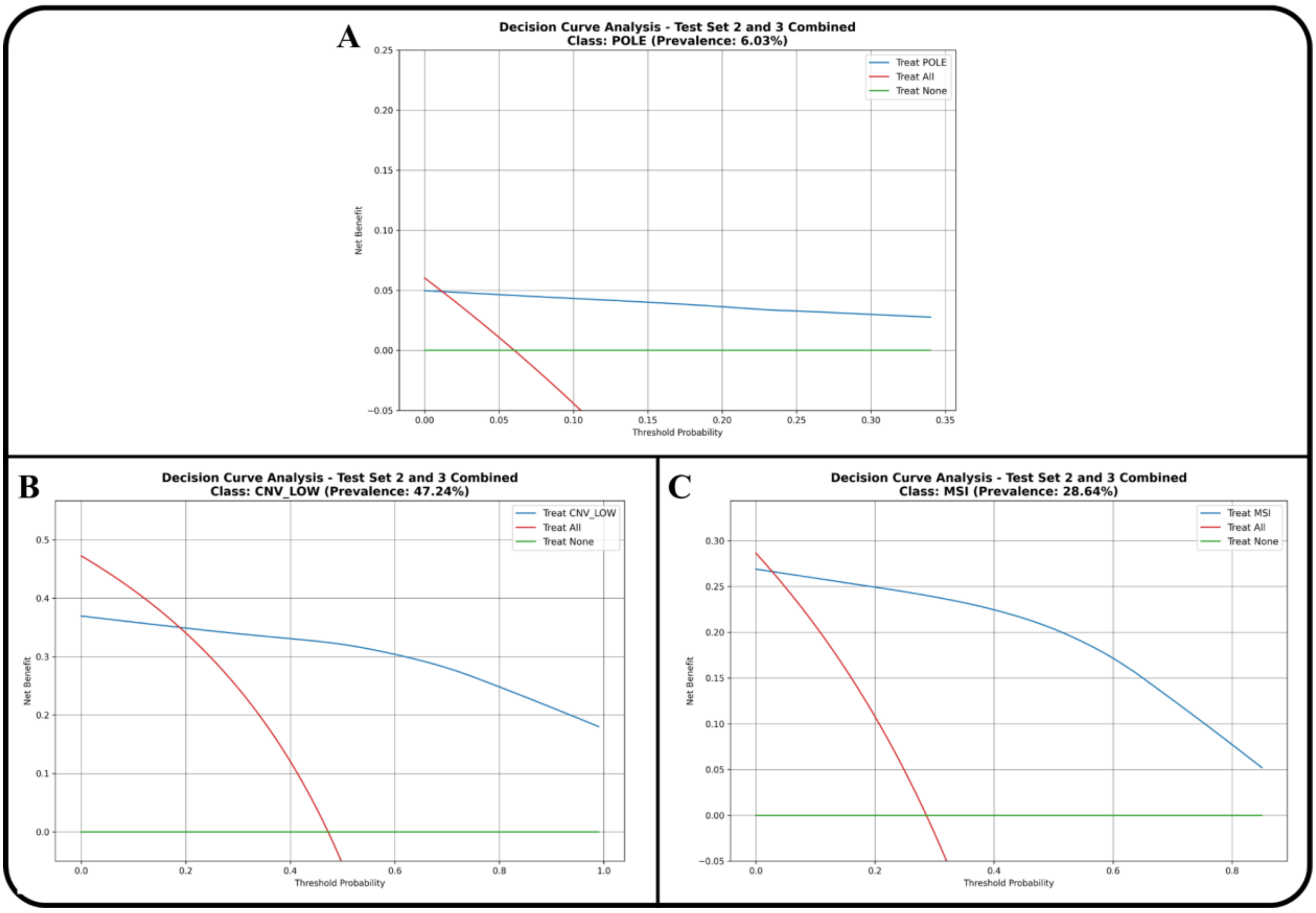

Supplementary Figure 3. Decision curve analysis for genomic subtype classification across molecular subtypes on combined external test sets (Set-2 and Set-3). *Net benefit of the MLP model is shown for (A) POLE (prevalence: 6.03%), (B) CNV-Low (prevalence: 47.24%), and (C) MSI (prevalence: 28.64%) subtypes across a range of threshold probabilities. In each panel, the model-guided strategy (blue) is compared against treat-all (red) and treat-none (green) reference strategies.*

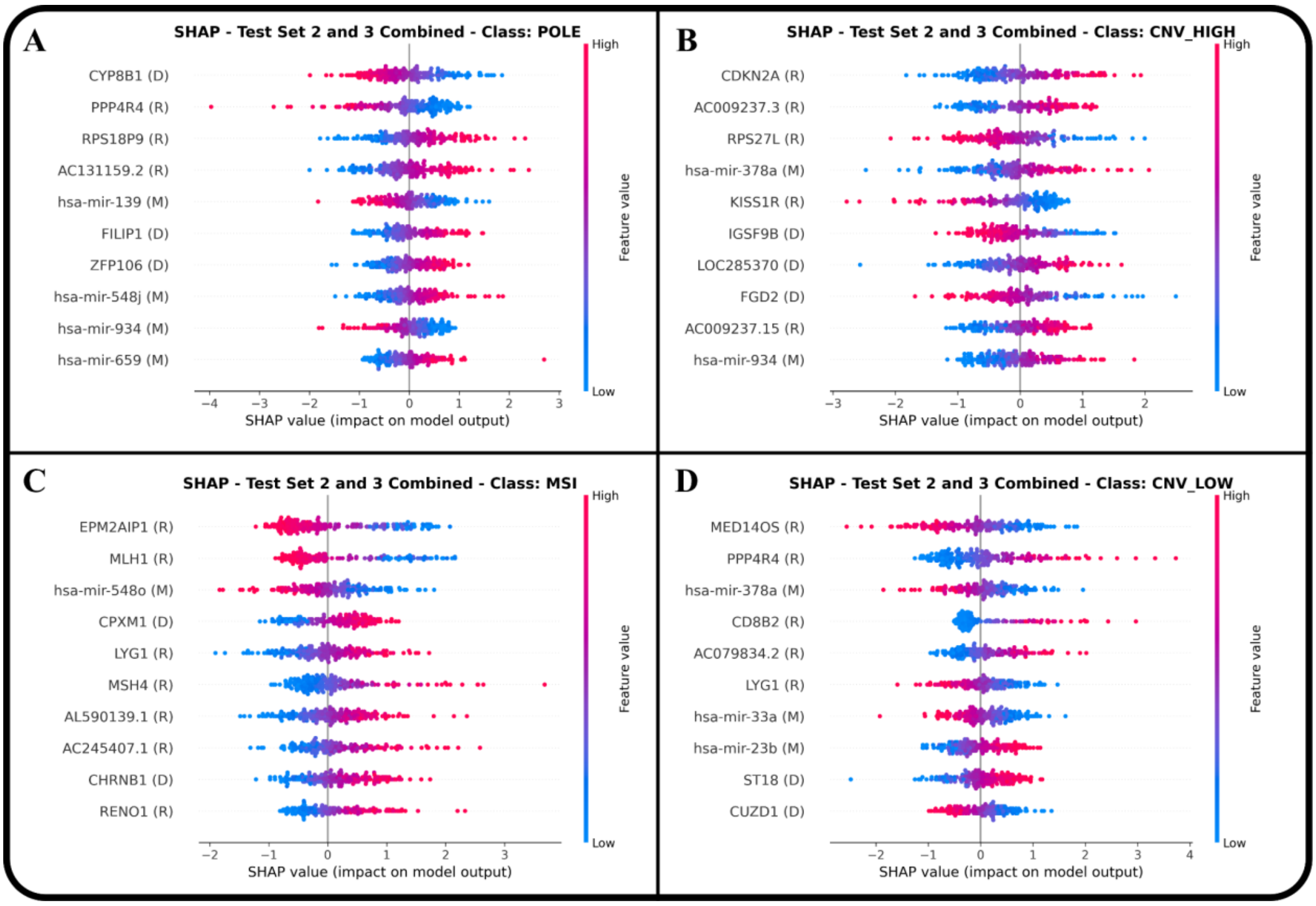

Supplementary Figure 4. SHAP-based feature importance analysis for the genomic subtype model on the combined external test tests (Set-2 and Set-3). *Beeswarm plots depicting the top 10 most influential features identified by SHAP (SHapley Additive exPlanations) analysis for each of the four endometrial cancer molecular subtypes: (A) POLE, (B) CNV-High, (C) MSI, and (D) CNV-Low. Point colour reflects the relative feature value, ranging from low (blue) to high (red). Feature labels indicate the data modality of origin: RNA-seq expression (R), DNA methylation (M), or DNA methylation (D).*

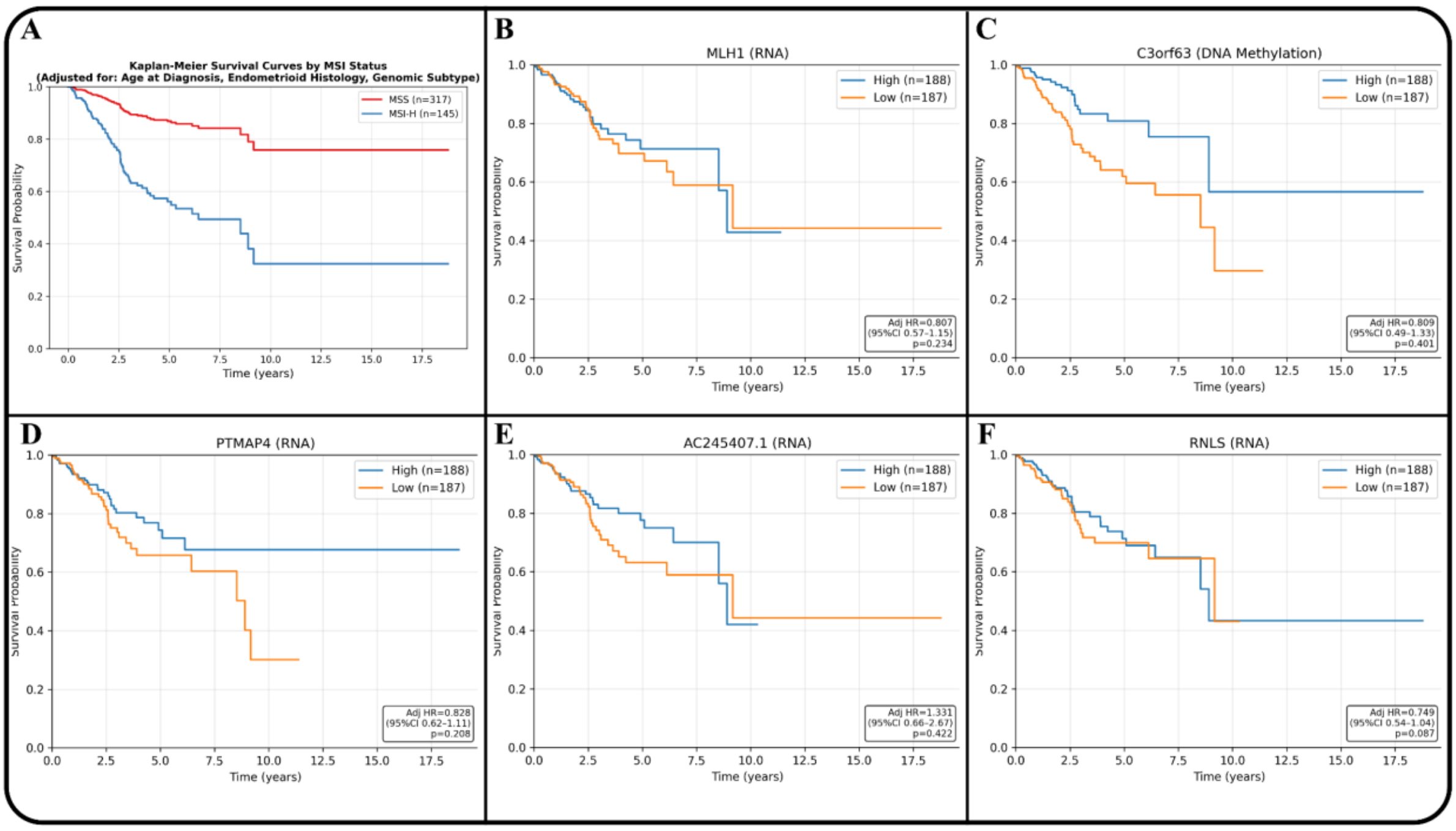

Supplementary Figure 5. Survival analysis for MSI-H versus MSS classification and selected molecular features. *(A) Kaplan–Meier overall survival curves stratified by microsatellite instability status (MSI-H vs MSS). (B–F) Kaplan–Meier curves showing overall survival according to high and low levels of selected RNA and DNA methylation features: (B) MLH1 (RNA), (C) C3orf63 (DNA methylation), (D) PTMAP4 (RNA), (E) AC245407.1 (RNA), and (F) RNLS (RNA). Patients were dichotomized into high and low groups using median values. Hazard ratios (HR) are shown for each feature.*

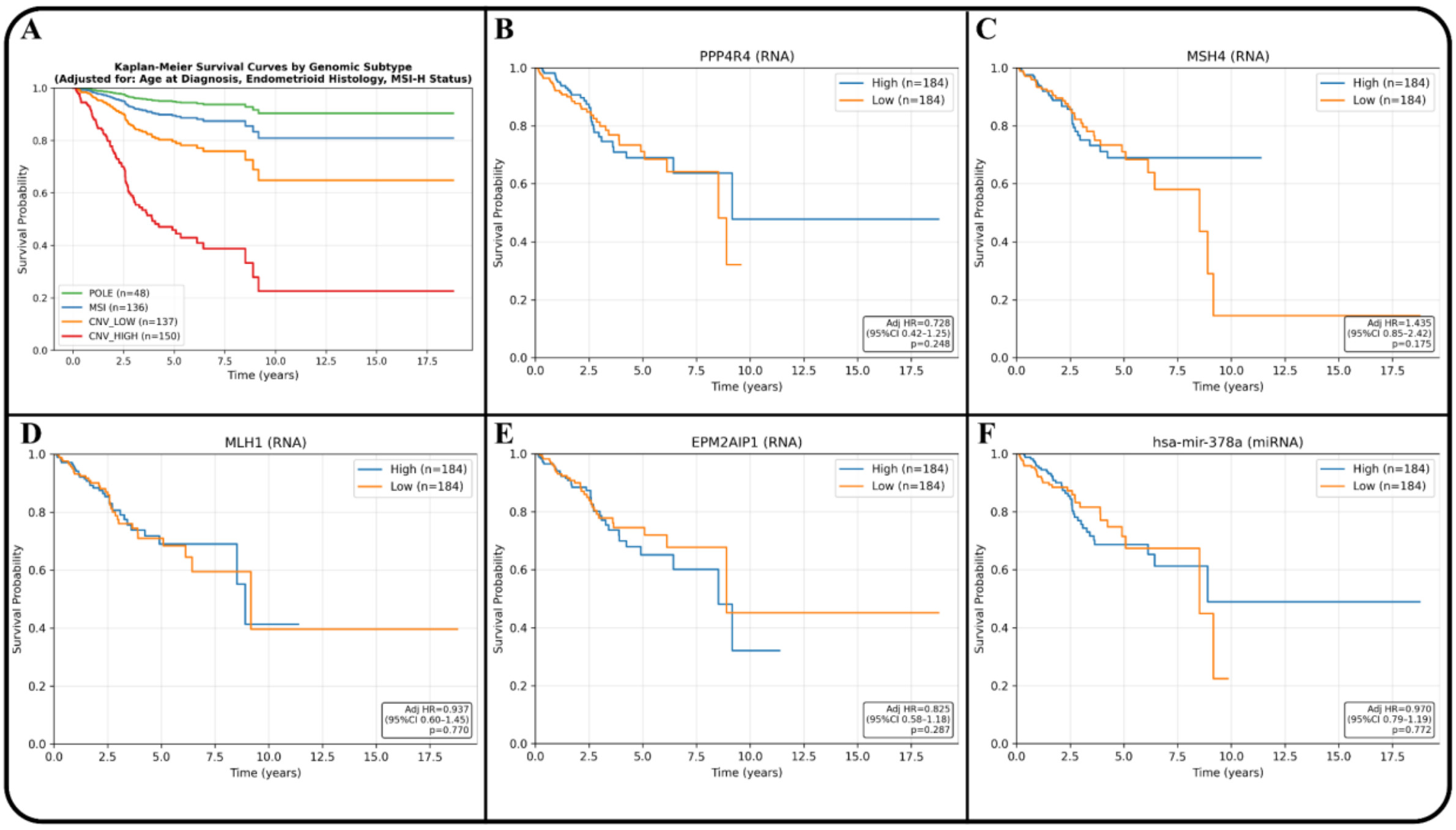

Supplementary Figure 6: Survival analysis by genomic subtype and selected molecular features. *(A) Kaplan–Meier overall survival curves stratified by genomic subtypes (CNV_HIGH, CNV_LOW, MSI, and POLE). (B–F) Kaplan–Meier curves showing overall survival according to high and low expression levels of selected RNA and microRNA features: (B) PPP4R4, (C) MSH4, (D) MLH1, (E) EPM2AIP1, and (F) hsa-miR-378a. Patients were dichotomized into high and low expression groups based on median values. Hazard ratios (HR) are shown for each feature.*

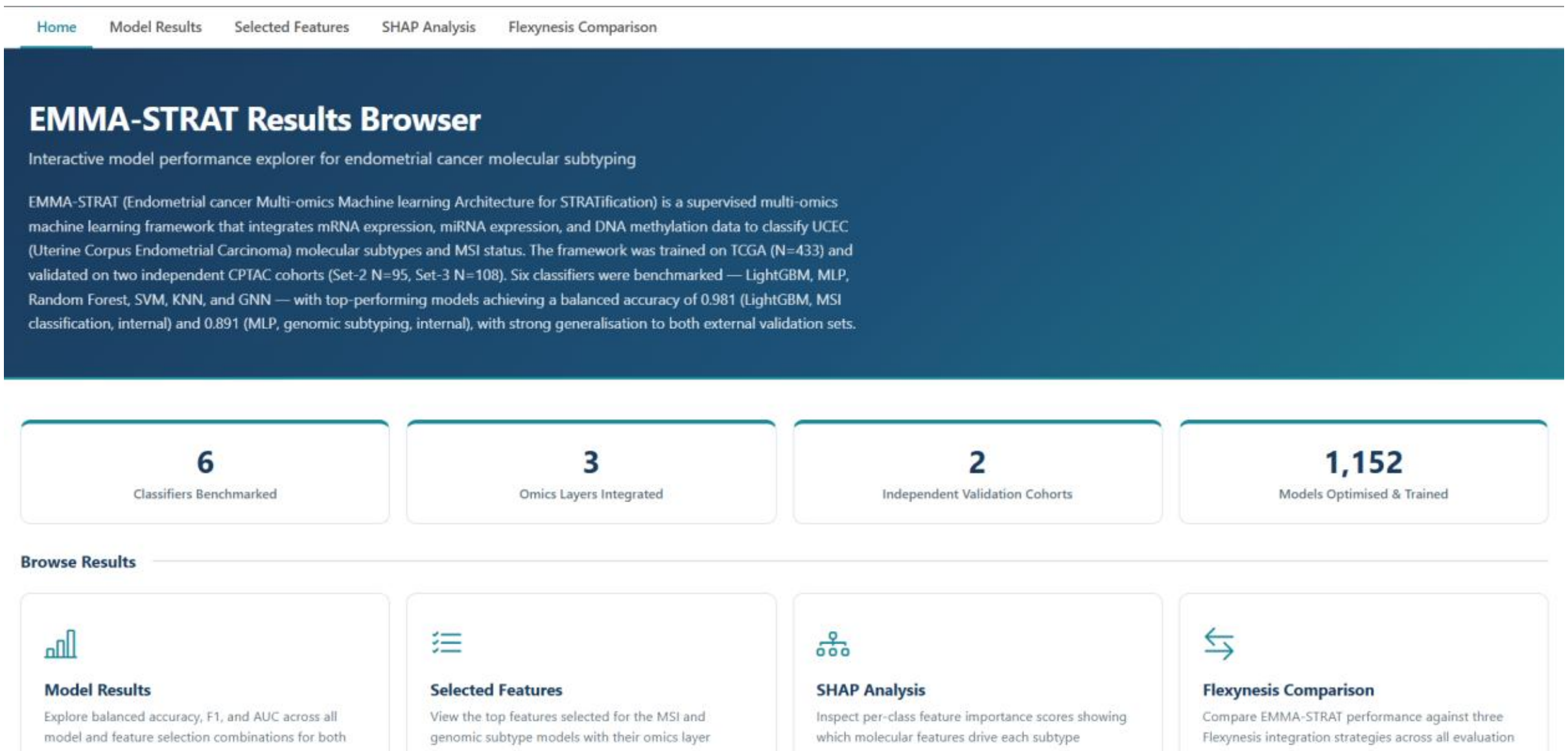

Supplementary Figure 7. *Screenshot of the EMMA-STRAT interactive results browser home page, accessible at https://naisarg14.github.io/EMMA-STRAT-web-viewer/index.html. The landing page provides a summary of the framework, key statistics, and navigation cards linking to the four browser sections: Model Results, Selected Features, SHAP Analysis, and Flexynesis Comparison.*

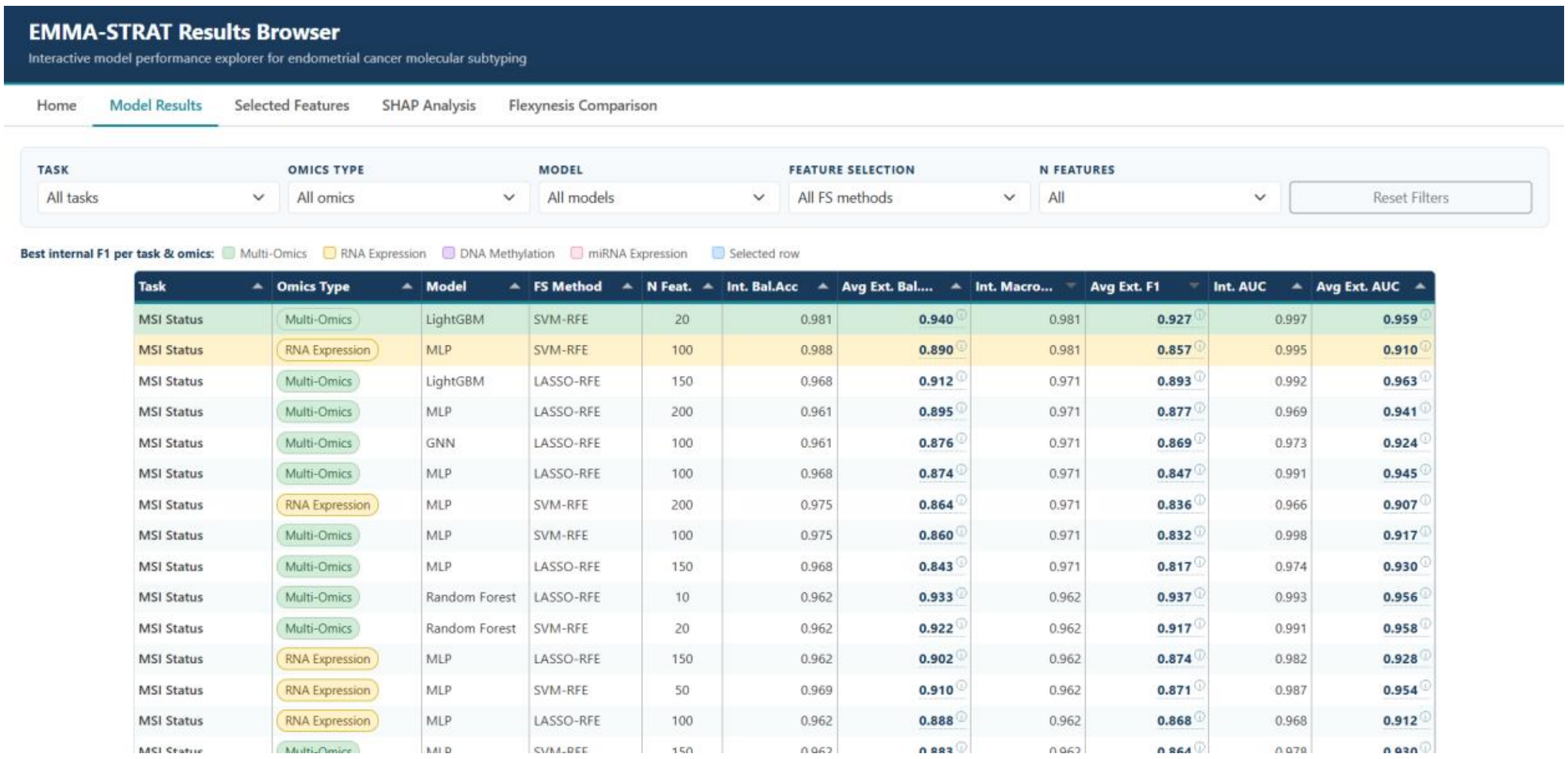

Supplementary Figure 8. *Screenshot of the Model Results page of the EMMA-STRAT interactive results browser [https://naisarg14.github.io/EMMA-STRAT-web-viewer/results.html]. Results are filterable by classification task, omics type, model architecture, feature selection method, and feature count. For each configuration, internal balanced accuracy, average external balanced accuracy, internal and average external macro F1-score, and internal and average external AUC are displayed. Rows corresponding to the best internal macro F1-score per task and omics type are colour-highlighted.*

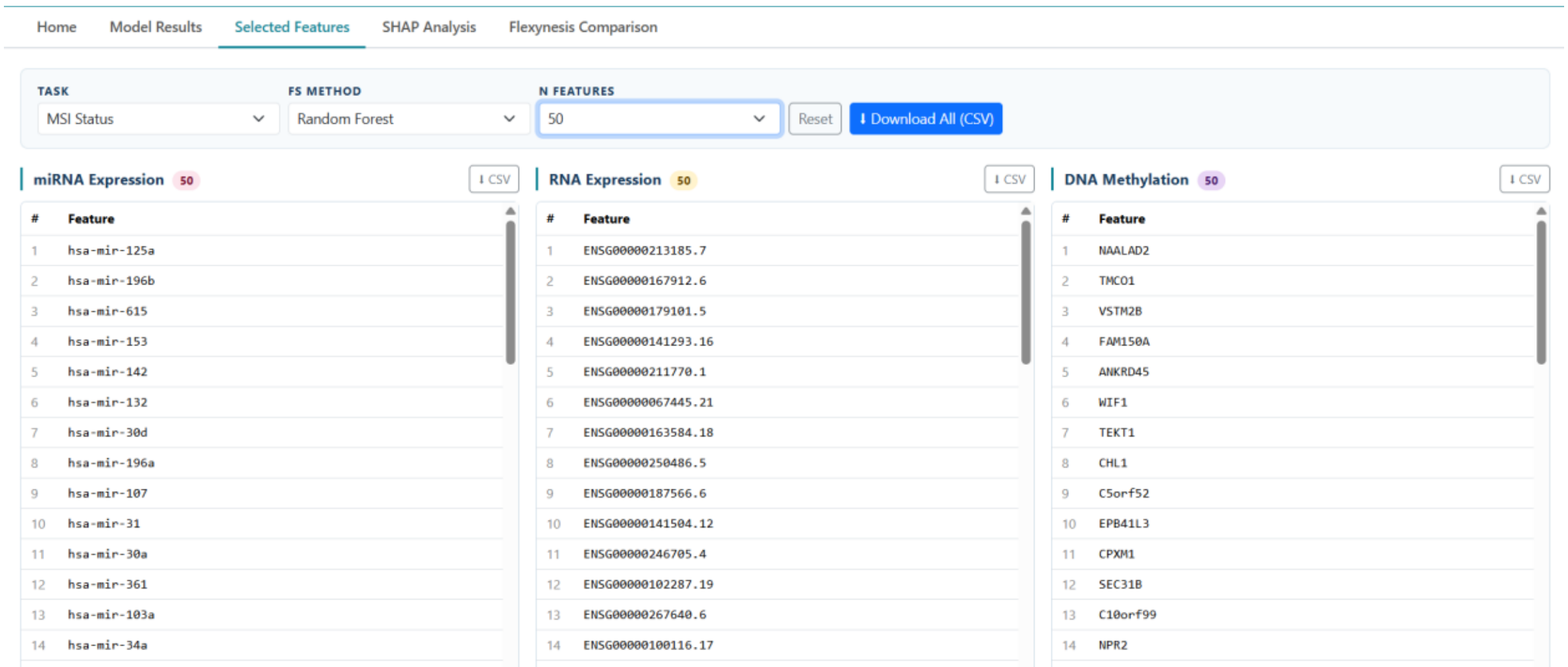

Supplementary Figure 9. *Screenshot of the Selected Features page of the EMMA-STRAT interactive results browser [https://naisarg14.github.io/EMMA-STRAT-web-viewer/features.html]. Features are displayed separately for each omics layer (miRNA expression, RNA expression, and DNA methylation) and are filterable by classification task, feature selection method, and feature count. Individual omics-layer feature lists are available for download as CSV files.*

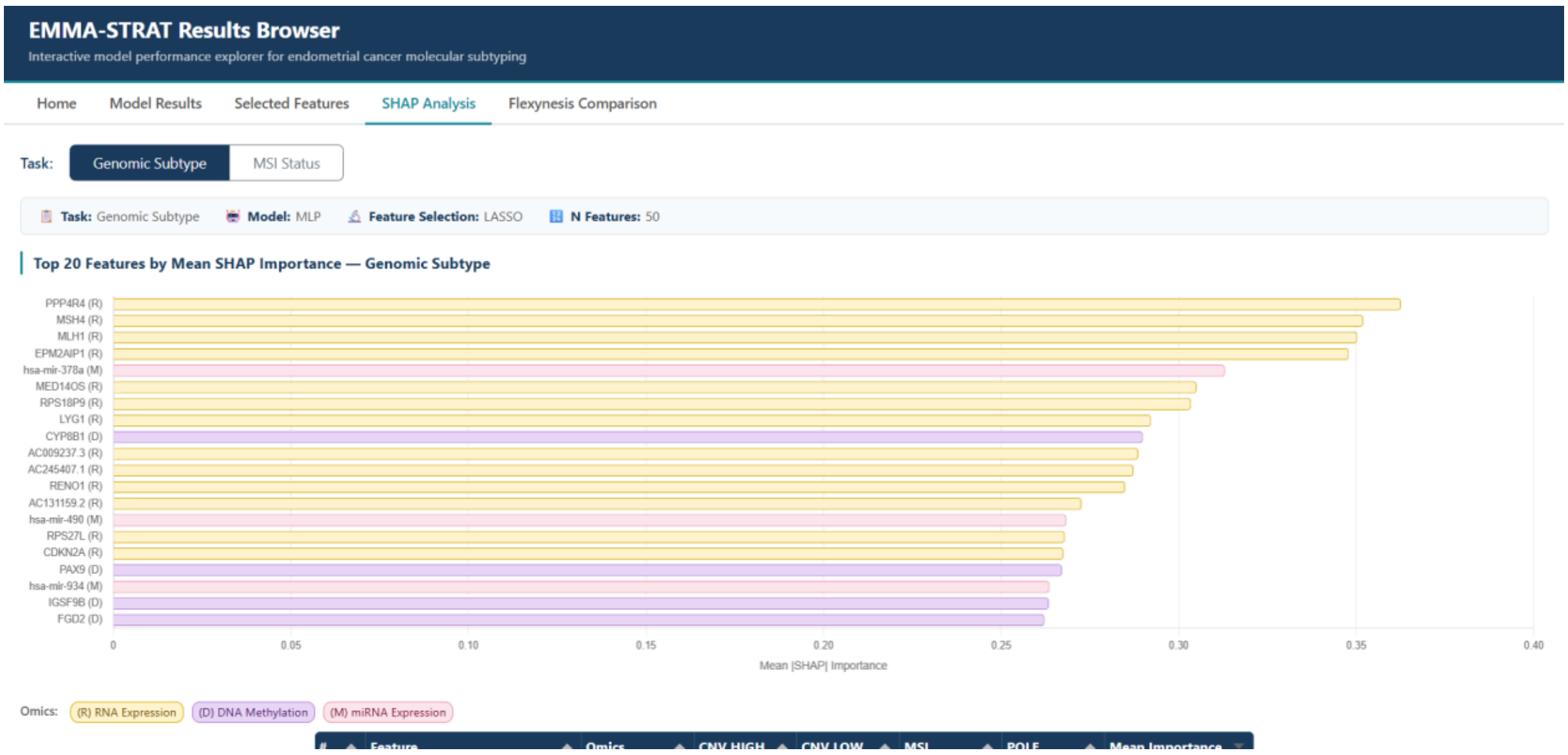

Supplementary Figure 10. *Screenshot of the SHAP Analysis page of the EMMA-STRAT interactive results browser, shown here for the genomic subtype classification task (MLP, LASSO-RFE, 50 features) [https://naisarg14.github.io/EMMA-STRAT-web-viewer/shap.html]. The top 20 features are displayed as a horizontal bar chart of mean absolute SHAP importance, colour-coded by omics layer (RNA expression, DNA methylation, miRNA expression). A sortable table below the chart reports per-class SHAP importance scores alongside mean importance for each feature. Results can be toggled between the genomic subtype and MSI status models.*

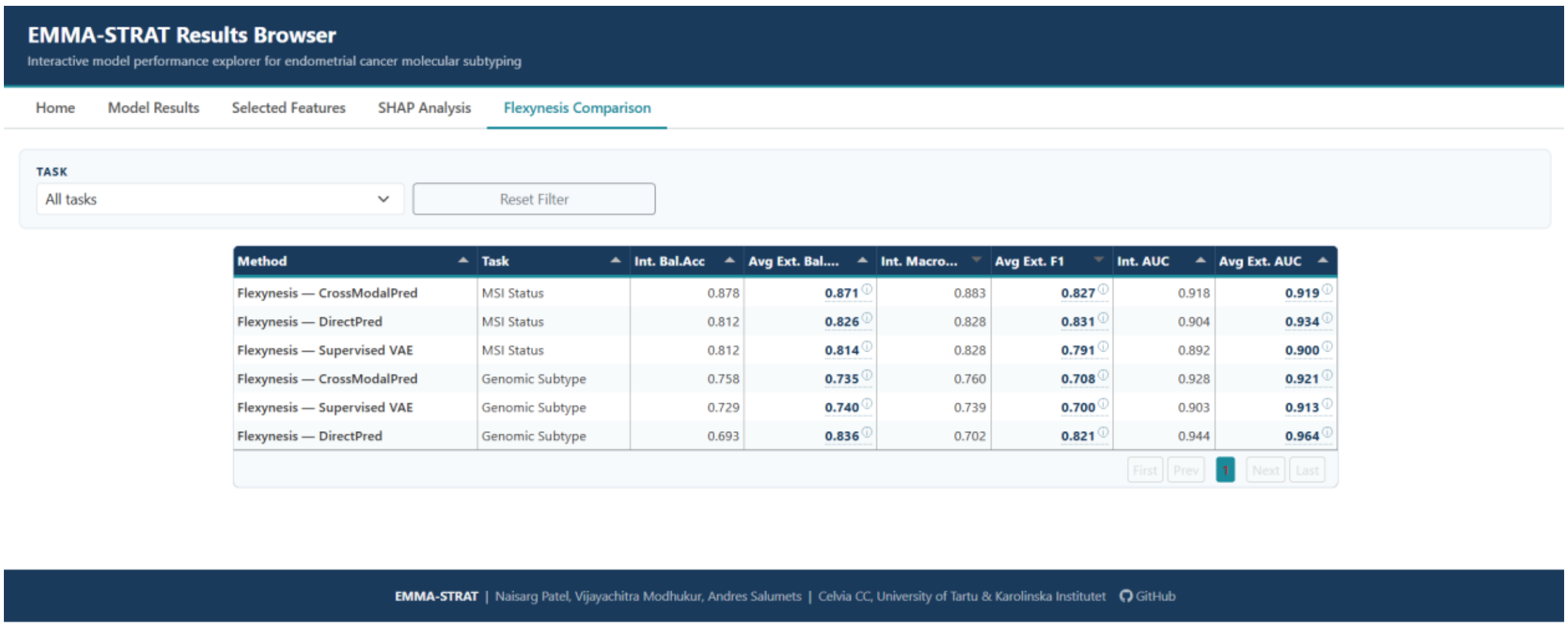

Method	Task	Int. Bal.Acc	Avg Ext. Bal....	Int. Macro...	Avg Ext. F1	Int. AUC	Avg Ext. AUC
Flexynesis — CrossModalPred	MSI Status	0.878	**0.871**	0.883	**0.827**	0.918	**0.919**
Flexynesis — DirectPred	MSI Status	0.812	**0.826**	0.828	**0.831**	0.904	**0.934**
Flexynesis — Supervised VAE	MSI Status	0.812	**0.814**	0.828	**0.791**	0.892	**0.900**
Flexynesis — CrossModalPred	Genomic Subtype	0.758	**0.735**	0.760	**0.708**	0.928	**0.921**
Flexynesis — Supervised VAE	Genomic Subtype	0.729	**0.740**	0.739	**0.700**	0.903	**0.913**
Flexynesis — DirectPred	Genomic Subtype	0.693	**0.836**	0.702	**0.821**	0.944	**0.964**

Supplementary Figure 11. *Screenshot of the Flexynesis Comparison page of the EMMA-STRAT interactive results browser [https://naisarg14.github.io/EMMA-STRAT-web-viewer/flexynesis.html]. The table presents internal balanced accuracy, average external balanced accuracy, internal and average external macro F1-score, and internal and average external AUC for all three Flexynesis integration strategies (DirectPred, Supervised VAE, CrossModalPred) across both classification tasks, filterable by task.*
